# Supplementary material for: Insights into Kinesin-1 Activation from the Crystal Structure of KLC2 Bound to JIP3
Source: Structure. 2018 Nov 6;26(11):1486–1498.e6. doi: 10.1016/j.str.2018.07.011 (PMC6224480; doi:10.1016/j.str.2018.07.011)
Supplement: Document S1. FiguresS1 and S2 and Tables S1–S5 [file mmc1.pdf]

Structure, Volume *26*

Supplemental Information

Insights into Kinesin-1 Activation

from the Crystal Structure of KLC2 Bound to JIP3

Joseph J.B. Cockburn, Sophie J. Hesketh, Peter Mulhair, Maren Thomsen, Mary J. O'Connell, and Michael Way

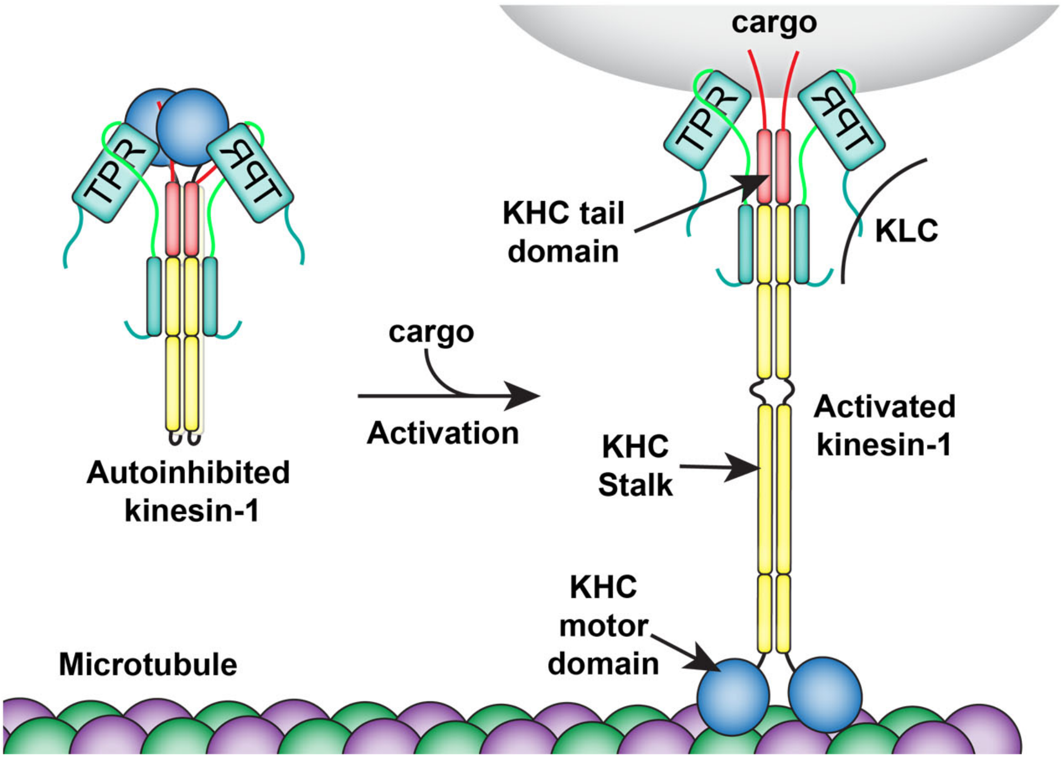

Figure S1

Figure S1. Related to Figure 1. Overview of kinesin and JIP3

Schematic of the kinesin heterotetramer in the autoinhibited and activated states.

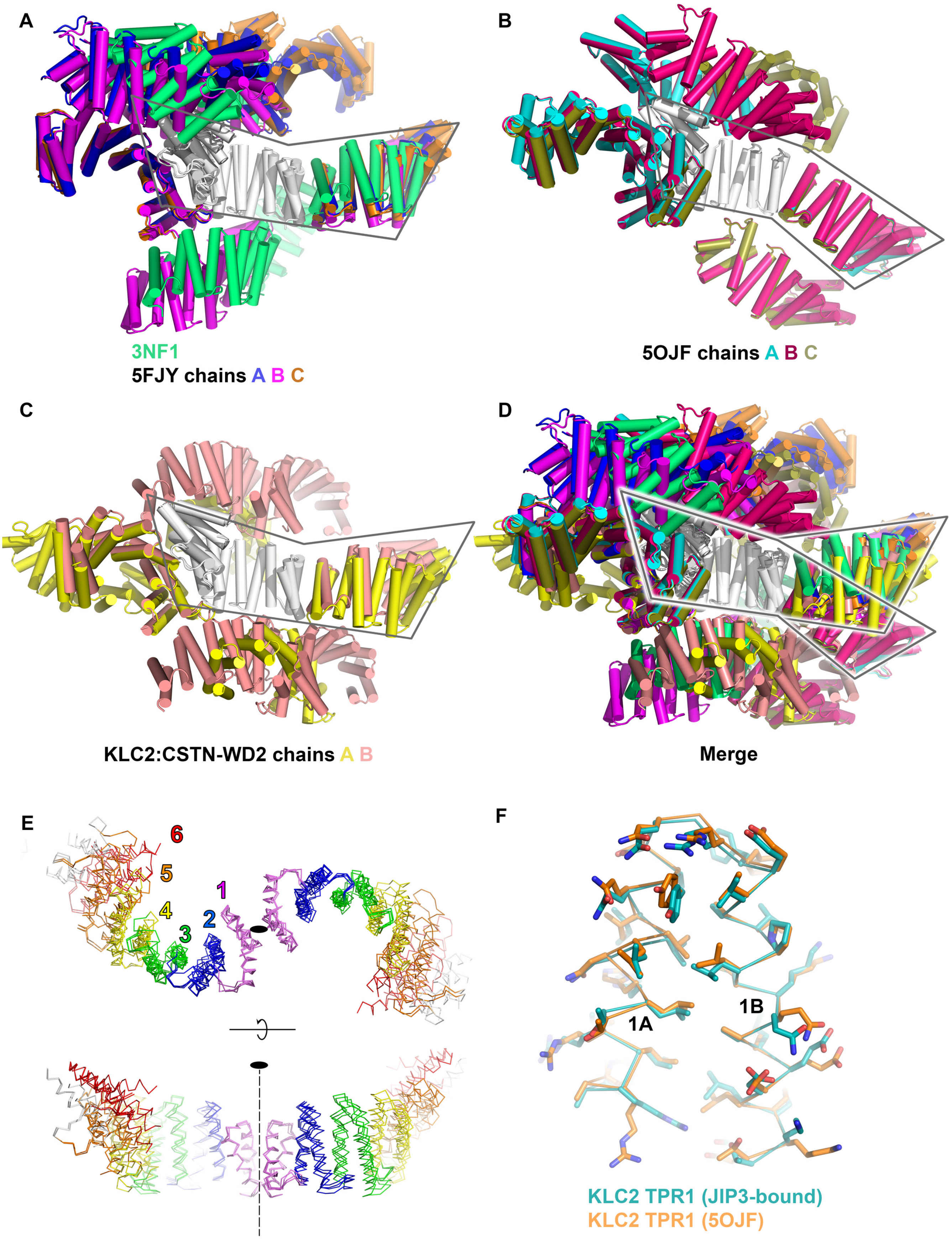
A
3NF1
5FJY chains A B C
B
5OJF chains A B C
C
KLC2:CSTN-WD2 chains A B
D
Merge
E
6
5
4
3
2
1
F
1A
1B
KLC2 TPR1 (JIP3-bound)
KLC2 TPR1 (5OJF)

Figure S2

Figure S2. Related to Figure 6. Dimerisation of KLC TPR domains via TPR1.

(**A-D**) Crystal packing interactions in (**A**) 3NF1 and 5FJY, (**B**) 5OJF, (**C**) our KLC2:CSTN-WD2 structure, and (**D**) all four structures. The environments of the crystallographically independent subunits (white) are superposed, with neighbours of 5FJY chains A/B/C coloured blue/magenta/orange; neighbours of 3NF1 chain A - green; neighbours of 5OJF chains A/B/C coloured cyan/hot pink/olive; and neighbours of KLC2:CSTN-WD2 chains A/B - pink/yellow. The conserved dimers are outlined.

(**E**) All 4 dimers from 3NF1 5FJY and our KLC2:CSTN-WD2 structure, coloured by TPR, superposed via TPR1 (dimer 2-fold axis – dashed line with oval).

(F) Superposition of KLC2 TPR1 from our KLC2:JIP3 complex (teal) and KLC2 TPR1 from 5OJF (orange), demonstrating very similar main-chain and side-chain conformations in these two structures.

Table S1. Related to Table 2 and Fig. 2. Locally-fitted parameters for ITC experiments with KLC2^{TPR} and GST-JIP3^{LZ}

**Expt.** [a]	**Cell**	**Syringe**	**Nominal cell conc. (μM)**	**Nominal syringe conc. (μM)**	**Fitted cell conc. (μM)**	**Fitted syringe conc. (μM)**	**Fitted baseline (kCal/mol)**
1	KLC2^{TPR}	GST-JIP3^{LZ}	38.0	332	36.5	392.5	0.45
2	KLC2^{TPR}	GST-JIP3^{LZ}	71	480	70.8	589.6	0
3	GST-JIP3^{LZ}	KLC2^{TPR}	47.0	418	49.5	481.5	0
4	GST-JIP3^{LZ}	KLC2^{TPR}	22.0	392	19.8	397.2	-0.32

[a] Results from experiments 1-4 are shown in Fig. 2A-D respectively

Table S2. Related to Fig. 3. Thermodynamics of the Trp-JIP3(LZ) domain and the FITC-CSTN-WD2 peptide binding to wild-type and mutant KLC2(TPR) domains

**KLC2 mutant**	**Ligand**	***K*** **(μM^{-1})**[a]	**ΔH (kCal/ mol)**	**$-T\Delta S$ (kCal/ mol)**	**Fitted concentration correction factors**[b]		**Fitted active fractions**[c]		**N_{app}**[d]	***n***[e]
					**KLC2**	**Ligand**	**KLC2**	**Ligand**		
WT	LZ	0.119[f]<br>0.129[g]	-19.667[f]<br>-4.719[g]	12.744[f]<br>-2.254[g]	0.98 ± 0.01	1.04 ± 0.06	1.0	1.0	N/A	3
R312E	LZ	0.229[f]<br>0.166[g]	-21.331[f]<br>-3.027[g]	14.019[f]<br>-4.093[g]	0.89 ± 0.08	0.99 ± 0.03	1.0	1.0	N/A	3
WT	CSTN	0.820	-4.305	-3.762	0.92 ± 0.18	1.32 ± 0.09	0.97	1.0	0.67 ± 0.08	2
Y208A	LZ	N/D[e]	N/D	N/D	N/D	N/D	N/D	N/D		2
Y208A	CSTN	0.541	-3.358	-4.463	0.98 ± 0.07	1.31 ± 0.04	0.90	1.0	0.67 ± 0.07	2
T200D	LZ	N/D	N/D	N/D	N/D	N/D	N/D	N/D		2
T200D	CSTN	0.845	-4.174	-3.911	0.96 ± 0.03	1.34 ± 0.03	1.0	1.0	0.71 ± 0.03	3
L220Y	LZ	N/D	N/D	N/D	N/D	N/D	N/D	N/D		2
L220Y	CSTN	0.739	-3.902	-4.104	0.92 ± 0.00	1.30 ± 0.02	1.0	1.0	0.71 ± 0.01	2

[a] Macroscopic association constant

[b] Locally-fitted correction factors for KLC2 and ligand concentrations during fitting of the relevant model (mean ± standard deviation of the locally fitted values in the *n* experiments)

[c] Active fractions of KLC2 or ligand (globally-fitted over the *n* experiments in the dataset during fitting of the relevant model)

[d] Apparent binding stoichiometry (see Methods; mean ± standard deviation of values from the *n* experiments)

[e] Number of experiments

[f,g] Parameters for $KLC2^{TPR}$ binding to the first and second sites on the Trp-$JIP3^{LZ}$ dimer, respectively.

[h] Not determined

Table S3. Related to Figure 5. Conservation of the JIP3 KLC-binding site, and KLC cargo binding sites, LFP motif, and TPR1 dimerisation site in Bilaterian species*

Species	KLC binding site on JIP3	JIP3 binding site on KLC	Tryptophan-acidic cargo binding site	KLC LFP motif	KLC TPR1 dimerisation interface
**Chordata**					
A. platyrhynchos	✓	✓	✓	✓	✓
A. carolinensis	✓	✓	✓	✓	✓
A. mexicanus	✓	✓	✓	✓	✓
B. floridae	✓	✓	✓	✓	✓
G. gallus	✓	✓	✓	✓	✓
C. hoffmanni	✓	✗	✗	✓	✗
C. intestinalis	✓	✓	✓	✗	✓
C. savignyi	✓	✓	✓	✗	✓
D. rerio	✓	✓	✓	✓	✓
D. novemcinctus	✓	✓	✓	✗	✓
E. telfairi	✓	✓	✓	✓	✓
F. albicollis	✓	✓	✓	✓	✓
G. morhua	✗	✓	✓	✓	✓
G. aculeatus	✓	✓	✓	✓	✓
H. sapiens	✓	✓	✓	✓	✓
L. chalumnae	✓	✓	✓	✓	✓
L. oculatus	✓	✓	✓	✓	✓
L. africana	✓	✓	✓	✓	✓
M. eugenii	✓	✓	✓	✓	✓
M. gallopavo	✓	✓	✓	✓	✓
M. domestica	✓	✓	✓	✓	✓
M. musculus	✓	✓	✓	✓	✓
M. lucifugus	✓	✓	✓	✓	✓
N. leucogenys	✓	✓	✓	✓	✓
O. niloticus	✓	✓	✓	✓	✓
O. anatinus	✓	✓	✓	✓	✓
O. latipes	✓	✓	✓	✓	✓
O. garnettii	✓	✓	✓	✓	✓
P. sinensis	✓	✓	✓	✓	✓
P. marinus	N/A	✓	✓	✓	✓
S. scrofa	✓	✓	✓	✓	✓
P. formosa	✓	✓	✓	✓	✓
P. capensis	✓	✓	✓	✓	✓
R. prolixus	N/A	✓	✓	✓	✓
S. harrisii	✓	✓	✓	✓	✓
S. araneus	✓	✓	✓	✗	✓
S. purpuratus	✓	✓	✓	✗	✓
T. guttata	✓	✓	✓	✓	✓

T. rubripes	✗	✓	✓	✓	✓
T. nigroviridis	✓	✓	✓	✓	✓
X. tropicalis	✓	✓	✓	✓	✓
X. maculatus	✓	✓	✓	✓	✓
**Arthropoda**					
A. aegypti	✗	✓	✓	✓	✓
A. gambiae	✓	✓	✓	✓	✓
D. plexippus	✓	✓	✓	✓	✓
D. pulex	✓	✓	✓	✓	✓
D. melanogaster	✓	✓	✓	✓	✓
N. vitripennis	N/A	✓	✓	✓	✓
S. maritima	✓	✓	✓	✗	✓
T. urticae	✓	✓	✓	✗	✓
T. castaneum	✓	✓	✓	✓	✓
Z. nevadensis	✓	✓	✗	✓	✓
**Nematoda**					
C. briggsae	✓	✓	✓	✗	✓
C. elegans	✓	✓	✓	✗	✓
O. volvulus	✓	✓	✓	✗	✓
**Annelida**					
C. teleta	✓	✓	✓	✗	✓
H. robusta	✓	✓	✓	✗	✓
**Mollusca**					
L. gigantea	✓	✓	✓	✗	✓
**Platyhelminthes**					✓
S. mansoni	✓	✓	✓	✗	✓

*Key to symbols:
✓ - conserved (see Materials and Methods)
✗ - not conserved
N/A - no homology detected

Table S4. Related to Figure 5. Conservation of the JIP3 KLC-binding site, and KLC cargo binding sites, LFP motif, and TPR1 dimerisation site in non-Bilaterian species*

Species	KLC binding site on JIP3	JIP3 binding site on KLC	Tryptophan-acidic cargo binding site	KLC LFP motif	KLC TPR1 dimerisation interface
**Ichthyosporea**					
A. whisleri	N/A	N/A	✗	N/A	N/A
C. fragrantissima	N/A	N/A	N/A	N/A	N/A
P._gemmata	N/A	N/A	N/A	N/A	N/A
**Porifera**					
A. queenslandica	N/A	✗	✗	✗	✗
A. vastus	✗	✗	✗	✗	✗
C. nucula	✗	N/A	✗	✗	✗
C. candelabrum	✗	✓	✓	✗	✗
E. muelleri	✗	✗	✗	✗	✗
G. compressa	✗	✓	✗	✗	✓
I. fasciculata	N/A	✗	✗	✗	✗
L. nivea	✗	✓	✗	✗	✓
O. carmela	✗	✗	✗	N/A	✗
P. ficiformis	✗	✗	✗	✗	✗
P. jani	✗	✓	✓	N/A	✗
P. spinifera	✗	✗	✗	✗	✗
S. lacustris	N/A	✗	✗	N/A	✗
S. coactum	✗	✓	✗	✗	✓
Ctenophora					
B. abyssicola	N/A	✓	✗	✗	✓
B. infundibulum	N/A	✗	✗	✗	✗
C. veneris	N/A	✗	✗	N/A	N/A
C. astericola	N/A	✗	✓	N/A	✗
C. meteoris	N/A	✓	✓	✗	✓
Ctenophora_sp3_A	N/A	N/A	N/A	N/A	N/A
D. glandiformis	✗	✗	✗	✗	✗
E. dunlapae	N/A	✓	✗	✗	✓
L. pancerina	N/A	✓	✓	✗	✓
M. leidyi_A	N/A	✓	✗	✗	✓
M. leidyi	✗	✓	✓	✗	✓
P. pileus_A	N/A	✓	✓	N/A	✓
P. pileus	N/A	✓	✓	✗	✓
V. multiformis_A	N/A	N/A	N/A	N/A	N/A
V. multiformis	✗	✓	✓	✗	✓
**Cnidaria**					
A. caribbeana	✗	✓	✗	✗	✓
C. sowerbyi	N/A	✓	✓	✗	✓
H. polyclina	✗	✓	✗	✗	✓
L. tetraphylla	✗	✓	✓	✗	✓

L. campanulata	N/A	✓	✗	✗	✓
M. auretenra	N/A	✓	✗	N/A	✓
N. vectensis	✗	✓	✓	✗	✓
P. noctiluca	N/A	✓	✗	N/A	✓
P. rubra	N/A	N/A	N/A	N/A	N/A
P. pennacea	✗	✓	✗	N/A	✗

*Key to symbols:
✓ - conserved (see Materials and Methods)
✗ - not conserved
N/A - no homology detected

Table S5. Related to Figure 6. Summary of crystal structures containing full-length KLC TPR domain

Construct	Crystallisation conditions	Space group	Unit cell parameters a, b, c (Å) α, β, γ (°)	# copies[1]	PDB entry
**murine KLC2 (191-480) + CSTN-WD2 peptide**	0.893 M sodium potassium tartrate<br>0.2M sodium chloride<br>0.1 M imidazole pH 8.0	P 3_1 2 1	75.44, 75.44, 303.36<br>90, 90, 120	2	6EJO
**murine KLC2 (161-480)**	0.1 M sodium cacodylate, pH 6.5<br>0.3 M sodium malonate<br>8% (wt/vol) PGA-LM.	C2	148.70, 86.28, 111.74<br>90, 98.4, 90	3	5FJY
**human KLC1 (205-495)[2]**	2.0 M ammonium sulfate<br>20% ethylene glycol<br>0.1 M Bis-Tris Propane pH 7.0	P 3_1 2 1	74.7, 74.7, 156.2<br>90, 90, 120	1	3NF1
**murine KLC2 (194-479)**	0.5 M sodium postassium phosphate pH 7.5<br>12% peg 3350<br>1 mM sarcosine	C2	97.62, 116.47, 108.11<br>90, 99.51, 90	3	5OJF

[1] per crystallographic asymmetric unit
[2] equivalent to murine KLC2 residues 190-480
