# Supplementary material for: Insights into Kinesin-1 Activation from the Crystal Structure of KLC2 Bound to JIP3
Source: Structure. 2018 Nov 6;26(11):1486–1498.e6. doi: 10.1016/j.str.2018.07.011 (PMC6224480; doi:10.1016/j.str.2018.07.011)
Supplement: Document S2. Article plus Supplemental Information [file mmc2.pdf]

Structure

Article

Insights into Kinesin-1 Activation from the Crystal Structure of KLC2 Bound to JIP3

Highlights

- Crystal structure of the kinesin-1 KLC2 TPR domain bound to the cargo JIP3
- JIP3 binding site is located on KLC TPR1 and is conserved from sponges to humans
- Molecular mechanism by which ARF6 regulates kinesin-1 recruitment by JIP3/4 revealed
- A unified framework explaining how unrelated cargoes activate kinesin-1

Authors

Joseph J.B. Cockburn,
Sophie J. Hesketh, Peter Mulhair,
Maren Thomsen, Mary J. O'Connell,
Michael Way

Correspondence

j.j.b.cockburn@leeds.ac.uk

In Brief

Cockburn et al. present the structure of the cargo binding TPR domain of kinesin light chain bound to the cargo molecule JIP3. From this they propose a mechanism by which multiple, unrelated cellular cargo molecules can "hotwire" the kinesin-1 molecule into motility.

Cockburn et al., 2018, Structure *26*, 1486–1498
November 6, 2018 © 2018 The Authors. Published by Elsevier Ltd.
https://doi.org/10.1016/j.str.2018.07.011

Structure
Article

Insights into Kinesin-1 Activation from the Crystal Structure of KLC2 Bound to JIP3

Joseph J.B. Cockburn,[1,4,*] Sophie J. Hesketh,[1] Peter Mulhair,[2] Maren Thomsen,[1] Mary J. O'Connell,[2] and Michael Way[3]
[1]Astbury Centre for Structural Molecular Biology, University of Leeds, Leeds LS2 9JT, UK
[2]Computational and Molecular Evolutionary Biology Research Group, Faculty of Biological Sciences, University of Leeds, Leeds LS2 9JT, UK
[3]Cellular Signalling and Cytoskeletal Function Laboratory, The Francis Crick Institute, 1 Midland Road, London NW1 1AT, UK
[4]Lead Contact
*Correspondence: j.j.b.cockburn@leeds.ac.uk
https://doi.org/10.1016/j.str.2018.07.011

SUMMARY

Kinesin-1 transports numerous cellular cargoes along microtubules. The kinesin-1 light chain (KLC) mediates cargo binding and regulates kinesin-1 motility. To investigate the molecular basis for kinesin-1 recruitment and activation by cargoes, we solved the crystal structure of the KLC2 tetratricopeptide repeat (TPR) domain bound to the cargo JIP3. This, combined with biophysical and molecular evolutionary analyses, reveals a kinesin-1 cargo binding site, located on KLC TPR1, which is conserved in homologs from sponges to humans. In the complex, JIP3 crosslinks two KLC2 TPR domains via their TPR1s. We show that TPR1 forms a dimer interface that mimics JIP3 binding in all crystal structures of the unbound KLC TPR domain. We propose that cargo-induced dimerization of the KLC TPR domains via TPR1 is a general mechanism for activating kinesin-1. We relate this to activation by tryptophan-acidic cargoes, explaining how different cargoes activate kinesin-1 through related molecular mechanisms.

INTRODUCTION

Kinesin-1 transports a wide variety of cellular cargoes toward the plus-ends of microtubules, including proteins, vesicles, mRNP complexes, and organelles, and is implicated in a number of diseases including neurodegeneration, viral and bacterial infections, and cancer (Dodding and Way, 2011; Hirokawa et al., 2009). The simplest form of kinesin-1 is the kinesin heavy chain (KHC) homodimer (Diefenbach et al., 2002; Glater et al., 2006; Su et al., 2004; Sun et al., 2011; Vale, 2003). In animal cells, kinesin-1 exists predominantly as a heterotetramer, which consists of the KHC homodimer bound to two copies of kinesin light chain (KLC) (Figure S1) (Gindhart et al., 1998; Johnson et al., 1990; Rahman et al., 1999). KHC comprises an N-terminal motor domain, a central coiled-coil stalk, and a C-terminal tail domain. KLC comprises an N-terminal heptad repeat region that oligomerizes with the KHC stalk, an acidic linker region, and a tetratricopeptide repeat (TPR) domain containing six TPRs (TPR1-6) (Figure 1A) (Gauger and Goldstein, 1993; Verhey et al., 1998; Wong and Rice, 2010; Zhu et al., 2012). The mammalian genome contains four KLC genes (KLC1-4). KLC2 is ubiquitously expressed, while other KLCs are enriched in certain tissues (Junco et al., 2001; Rahman et al., 1998).

The KHC motor domain hydrolyses ATP to power microtubule-based motility. At the other end of the molecule, the KHC tail domain and KLC harbor binding sites for cargoes (Blasius et al., 2007; Bowman et al., 2000; Byrd et al., 2001; Diefenbach et al., 2002; Fu and Holzbaur, 2013; Gindhart et al., 2003; Hammond et al., 2008; Nguyen et al., 2005; Pernigo et al., 2013; Seiler et al., 2000; Su et al., 2004; Sun et al., 2011; Verhey et al., 2001; Watt et al., 2015; Zhu et al., 2012). The ATPase activity of the motor domains is tightly regulated by cargo binding. When not bound to cargo, kinesin-1 adopts a folded-up conformation wherein the cargo binding regions inhibit the motor domains (Figure S1) (Cai et al., 2007; Coy et al., 1999; Hackney et al., 1992, 2009; Hackney and Stock, 2000; Stock et al., 1999). The binding of cargoes to the KHC tail and/or KLC releases the motor domains, leading to activation of kinesin-1 motility (Blasius et al., 2007; Dodding et al., 2011; Friedman and Vale, 1999; Fu and Holzbaur, 2013; Sun et al., 2011; Watt et al., 2015). This regulatory autoinhibition mechanism prevents futile ATP hydrolysis and congestion of microtubule tracks by kinesin-1 motors that are not engaged in cargo transport.

The molecular mechanism of kinesin-1 autoinhibition is best understood for the KHC homodimer. Here, a conserved stretch of residues in one copy of the KHC tail (the IAK motif) locks the motor domains together, preventing their stable association with microtubules (Hackney et al., 2009; Hackney and Stock, 2000; Kaan et al., 2011; Stock et al., 1999). Autoinhibition of the kinesin-1 heterotetramer involves an additional, poorly understood role for KLC (Verhey et al., 1998). Fluorescence resonance energy transfer experiments on KHC homodimers and kinesin-1 heterotetramers in cells showed that the presence of KLC results in the motor domains being splayed apart, relative to their configuration in the KHC homodimer, which was confirmed by chemical crosslinking (Cai et al., 2007). *In vitro* binding studies using purified recombinant proteins also showed that KLC reduces the affinity for the KHC tail domains for both the motor domains and microtubules (Wong and Rice, 2010). Recent work has shown that the kinesin-1 molecule is regulated by an intramolecular interaction within the KLC molecule, in which the TPR domain binds to a conserved Leu-Phe-Pro (LFP) motif in the upstream acidic linker (Yip et al., 2016) (Figure 1A). Disrupting this interaction

This is an open access article under the CC BY license (http://creativecommons.org/licenses/by/4.0/).

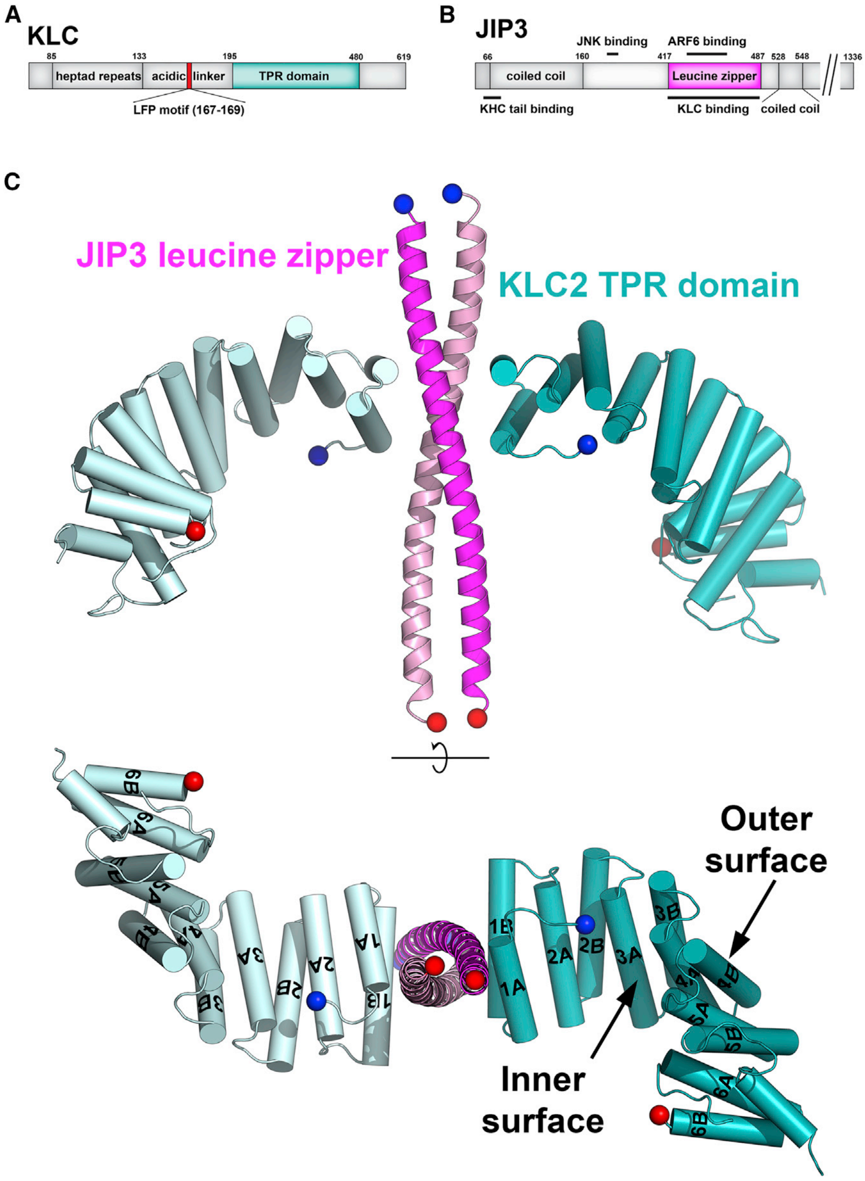

Figure 1. The Crystal Structure of the KLC2^{TPR}:JIP3^{LZ} Complex

(A and B) Schematics of the KLC2 (A) and JIP3 (B) molecules.

(C) The KLC2^{TPR}:JIP3^{LZ} complex. The KLC2^{TPR}s are shown in teal (chain A) and cyan (chain B). The KLC2^{TPR} α helices are shown as cylinders and labeled 1A through 6B. JIP3^{LZ} subunits are shown in magenta (chain C) and pink (chain D) cartoon. Blue/red spheres are N- and C-terminal C_{α} atoms, respectively.

See also Figure S1.

either through cargo binding or pharmacological inhibition destabilizes the autoinhibited state of the kinesin-1 molecule, with drastic effects on the organization of the microtubule cytoskeleton (Randall et al., 2017). Thus, KLC adds an additional layer of regulation to the kinesin-1 molecule, but its molecular basis is poorly understood. There is thus a major gap in our understanding of the predominant form of kinesin-1 found in animal cells.

The KLC TPR domain (KLC^{TPR}) is unusual in binding to multiple ligands using distinct sites (Hammond et al., 2008; Zhu et al., 2012). How are these distinct molecular recognition events integrated into a common pathway for activating kinesin-1? The molecular mechanisms by which cargoes bind to kinesin-1 and activate its motility are not well understood. Only one crystal structure of a kinesin-1:cargo complex has been published (Pernigo et al., 2013). Certain cargo molecules bind to the KLC^{TPR} using tryptophan-acidic motifs, which bind to the inner concave surface of the KLC^{TPR}, spanning TPRs 2–4 (Araki et al., 2007; Dodding et al., 2011; Kawano et al., 2012; Pernigo et al., 2013; Zhu et al., 2012). This releases the KLC LFP motif from its binding site on the TPR domain, resulting in kinesin-1 activation; although how release of the LFP motif is coupled to kinesin-1 activation has not been demonstrated (Yip et al., 2016).

Other kinesin-1 cargoes do not contain tryptophan-acidic motifs, yet the molecular details of how these cargoes bind to and activate kinesin-1 remain to be described. The c-Jun N-terminal kinase interacting protein 3 (JIP3) (Figure 1B) and the related protein JIP4 are adaptor molecules that mediate bidirectional transport of a variety of cargoes by linking them to kinesin-1 and dynein (Bowman et al., 2000; Byrd et al., 2001; Cavalli et al., 2005; Huang et al., 2011; Kelkar et al., 2000, 2005; Schulman et al., 2014; Verhey et al., 2001). Kinesin-1 recruitment by JIP3/4 is regulated by the GTPase ARF6, which binds to the JIP3/4 leucine zipper (LZ) domain (JIP3/4^{LZ}) and prevents KLC binding (Montagnac et al., 2009). Kinesin-1-driven motility of JIP3/4 is involved in numerous cellular processes and diseases including axonal outgrowth, transport and damage signaling; muscle development, endosomal trafficking, Huntington's disease, and cancer (Bowman et al., 2000; Byrd et al., 2001; Cavalli et al., 2005; Edwards et al., 2013; Marchesin et al., 2015; Montagnac et al., 2009; Morfini et al., 2009; Schulman et al., 2014; Watt et al., 2015). JIP3 activates kinesin-1 by relieving autoinhibition in two stages (Watt et al., 2015). The JIP3^{LZ} (Figure 1B) binds to the KLC^{TPR}, inducing an immotile microtubule-bound intermediate (Nguyen et al., 2005; Watt et al., 2015). Binding of the JIP3 N-terminal region to the KHC tails then triggers motility (Sun et al., 2017; Watt et al., 2015). JIP3 thus sequentially relieves KLC and KHC regulation of the kinesin-1 molecule, making it an ideal model cargo to study kinesin-1 activation.

Here, we describe the crystal structure of the KLC2 TPR domain (KLC2^{TPR}) bound to the JIP3^{LZ}. This reveals how ARF6

Table 1. X-Ray Data Collection and Refinement Statistics

	$KLC2^{TPR}$:$JIP3^{LZ}$	$KLC2^{TPR}$:CSTN-WD2
Data collection[a]		
Space group	$P\ 6_5$	$P\ 3_121$
Cell dimensions		
a, b, c (Å)	163.30, 163.30, 77.12	75.44, 75.44, 303.36
α, β, γ (°)	90.0, 90.0, 120.0	90.0, 90.0, 120.0
Resolution (Å)	81.65–3.20 (3.37–3.20)[b]	44.46–3.99 (4.21–3.99)
R_{sym}	0.105 (1.59)	0.095 (0.838)
I/σI	13.8 (1.6)	6.28 (1.4)
Completeness (%)	99.5 (99.8)	94.9 (95.9)
Redundancy	7.9 (8.0)	2.8 (2.8)
Refinement		
Resolution (Å)	67.71–3.20	44.46–3.99
No. of reflections	19,378	8,641
R_{work}/R_{free}	0.191/0.211	0.253/0.273
No. of atoms		
Protein	5,207	8,650
Ligand/ion	–	–
Water	–	–
B factors		
Protein	127.23	209
Ligand/ion		
Water		
RMSD		
Bond lengths (Å)	0.009	0.005
Bond angles (°)	1.12	0.976

RMSD, root-mean-square deviation.
[a]Data collected from one crystal in each case.
[b]Values in parentheses are for highest-resolution shell.

regulates kinesin-1 recruitment by JIP3/4. We show that JIP3 activates kinesin-1 by a different mechanism to tryptophan-acidic cargoes, and propose a unified framework explaining how unrelated cargoes activate kinesin-1 through related mechanisms.

RESULTS

The Crystal Structure of the $KLC2^{TPR}$:$JIP3^{LZ}$ Complex

To investigate the molecular mechanisms of kinesin-1 recruitment and activation by JIP3/4, and how this is regulated by ARF6, we determined the crystal structure of the murine KLC2 TPR domain ($KLC2^{TPR}$) in complex with the murine $JIP3^{LZ}$ to a resolution of 3.2 Å (Figure 1C; Table 1). The crystallographic asymmetric unit contains a single copy of the complex, which comprises two copies of $KLC2^{TPR}$ bound to one copy of the $JIP3^{LZ}$ dimer, which adopts a parallel coiled-coil conformation. The structures of the two $KLC2^{TPR}$s are essentially identical to each other and to those reported previously (Nguyen et al., 2017; Yip et al., 2016; Zhu et al., 2012). The six KLC TPRs stack into a solenoidal structure with a super-helical twist, generating an inner (concave) and outer (convex) surface (Figure 1C). The KLC^{TPR} binds $JIP3^{LZ}$ end-on, via TPR1. This was unanticipated, since TPR domain ligands usually bind to the concave inner surface (Zeytuni and Zarivach, 2012). As a result, the TPR domains wind away from the $JIP3^{LZ}$ when the complex is viewed down the 2-fold axis. The complex contains two non-overlapping, identical $KLC2^{TPR}$:$JIP3^{LZ}$ interfaces. At each interface, a single $KLC2^{TPR}$ (chain A or B in the PDB file) binds to both $JIP3^{LZ}$ subunits (chains C and D). The KLC2 TPR1 α helices pack flat against the side of the $JIP3^{LZ}$, lying across the $JIP3^{LZ}$ helices at a ~90° angle, and oriented with helix 1B proximal to the $JIP3^{LZ}$ N terminus (Figure 1C). This buries 571 $Å^2$ of surface area on each $KLC2^{TPR}$. Binding of $KLC2^{TPR}$ chains A/B buries 325 and 207 $Å^2$ on $JIP3^{LZ}$ chains C/D and D/C, respectively.

We studied binding of the $KLC2^{TPR}$ to the GST3C-$JIP3^{LZ}$ by isothermal titration calorimetry (ITC) (Figures 2A–2D). The data could not be globally fitted with a single-site binding model, showing that the two binding sites on the $JIP3^{LZ}$ dimer do not bind $KLC2^{TPR}$ independently. Therefore, we globally fitted a two-stage sequential binding model (A + B + B → BA + B → BAB, where A and B represent the GST3C-$JIP3^{LZ}$ dimer and $KLC2^{TPR}$, respectively) to the ITC data, which gave a good fit across the entire dataset (Figures 2A–2D; Tables S1 and 2). The macroscopic association constants for binding of the first and second $KLC2^{TPR}$ subunits were very similar, suggesting that binding displays positive cooperativity. Binding of the first $KLC2^{TPR}$ is enthalpically driven and involves a large, favorable enthalpy change, and a large, unfavorable entropy change, whereas binding of the second $KLC2^{TPR}$ is entropically driven and involves much smaller energetic changes. These observations suggest that binding of the first $KLC2^{TPR}$ induces conformational changes in the GST3C-$JIP3^{LZ}$. We therefore investigated the structure of the $JIP3^{LZ}$ using circular dichroism (CD) spectroscopy (Figure 2E). For these experiments, we used a $JIP3^{LZ}$ construct N-terminally fused to a tryptophan residue (Trp-$JIP3^{LZ}$) to facilitate protein concentration determination since $JIP3^{LZ}$ does not contain any Try or Trp residues. Analysis of the Trp-$JIP3^{LZ}$ CD spectra revealed a mean α-helical content of only 59% ± 3%, compared with 77% calculated for $JIP3^{LZ}$ from the $KLC2^{TPR}$:$JIP3^{LZ}$ crystal structure (Figure 2F). By comparison, CD spectra for $KLC2^{TPR}$-myc revealed a secondary structure content very similar to that calculated from the $KLC2^{TPR}$:$JIP3^{LZ}$ crystal structure (Figure 2G). The Trp-$JIP3^{LZ}$ CD spectra also displayed a deeper minimum at 208 nm relative to that at 222 nm (Figure 2E): the ratio of ellipticities at 222 and 208 nm was 0.88 ± 0.01 (n = 3), whereas coiled coils typically possess ratios greater than 1.0 (Zhou et al., 1994). Nonetheless, in size-exclusion coupled to multiple-angle laser light scattering (SEC-MALLS) experiments the $JIP3^{LZ}$ eluted as a single peak with a molecular weight of 15.9 kDa (Figure 2H), showing that the $JIP3^{LZ}$ is a stable dimer. The structure of the unbound Trp-$JIP3^{LZ}$ thus differs significantly from that observed in the $KLC2^{TPR}$:$JIP3^{LZ}$ crystal structure. These observations suggest that binding of the first $KLC2^{TPR}$ induces the $JIP3^{LZ}$ dimer to adopt the coiled-coil conformation, resulting in large energetic changes and promoting binding of the second $KLC2^{TPR}$ (Figure 2H).

The $JIP3^{LZ}$ Binds to KLC2 TPR1

The $KLC2^{TPR}$ binding site spans heptad repeats 2–5 (residues 433–435, 437–442, 444–446, and 450) of the $JIP3^{LZ}$ (Figure 3A).

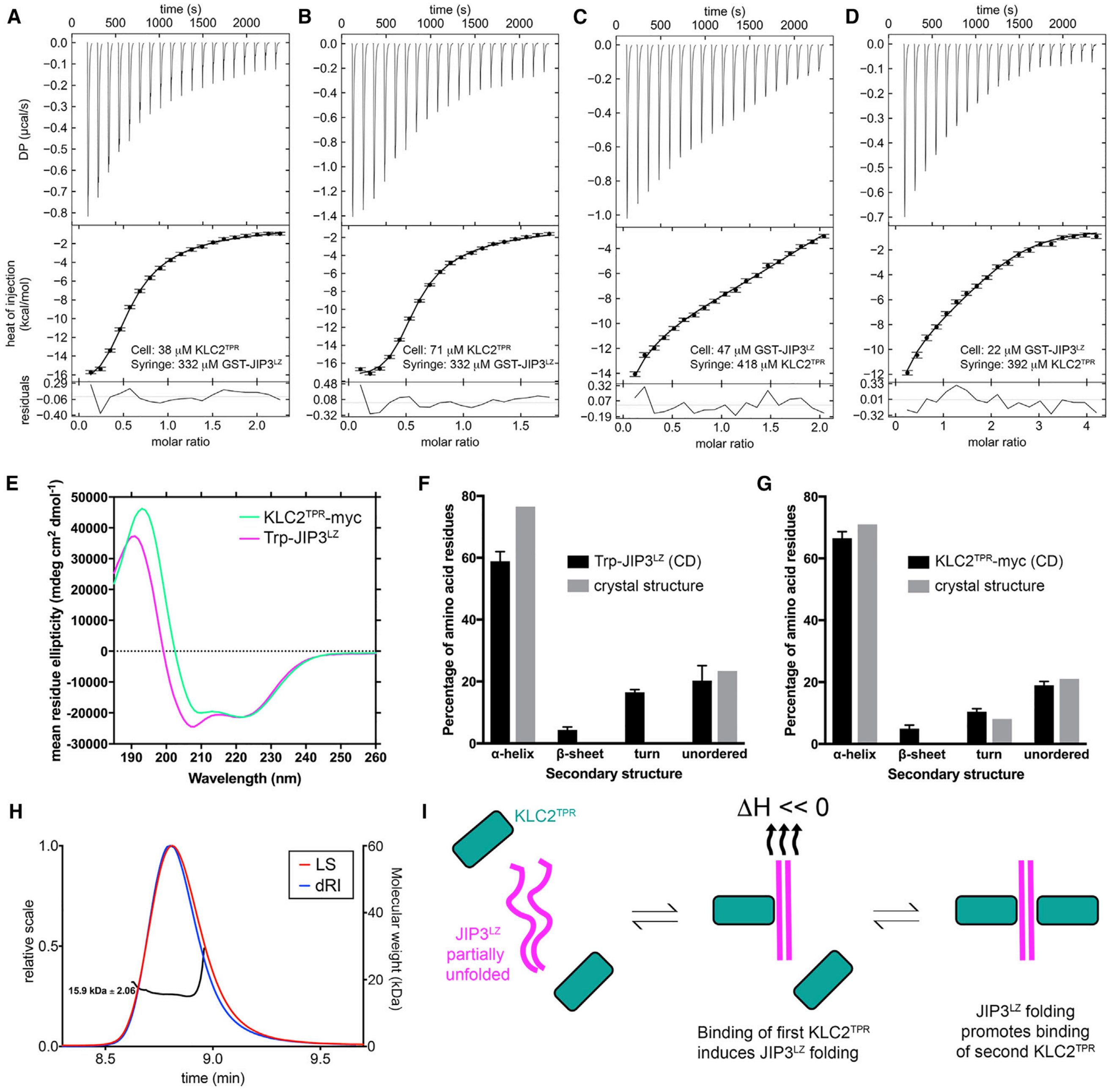

Figure 2. Biophysical Studies of $KLC2^{TPR}$ Binding to the $JIP3^{LZ}$

(A–D) ITC thermograms and isotherms for $KLC2^{TPR}$ binding to GST3C-$JIP3^{LZ}$. The error bars in the isotherms show the errors associated with integration of the injection peaks in the corresponding thermograms. Molar ratios correspond to the GST3C-$JIP3^{LZ}$ dimer concentration. The curves show the sequential binding model that was globally fitted across all the isotherms in the dataset. The lower panel shows the residuals between the isotherm data points and the fitted model.

(E) CD spectra of the Trp-$JIP3^{LZ}$ and $KLC2^{TPR}$-myc.

(F and G) Secondary structure composition of the Trp-$JIP3^{LZ}$ (F) and $KLC2^{TPR}$-myc (G). The black bars show the secondary structure composition determined from deconvolution of the CD spectra (mean and SD from three experiments). The gray bars show the Trp-$JIP3^{LZ}$ and $KLC2^{TPR}$-myc secondary structure compositions calculated from the $KLC2^{TPR}$:$JIP3^{LZ}$ crystal structure.

(H) SEC-MALLS chromatogram for the $JIP3^{LZ}$ domain showing the light scattering (LS) and differential refractive index (dRI) traces and molecular weight (black curve).

(I) Schematic showing the sequential binding model for $KLC2^{TPR}$ binding to the $JIP3^{LZ}$.

See also Tables 2 and S1.

Table 2. Globally Fitted Parameters for Sequential Binding of KLC2^{TPR} to GST-JIP3^{LZ}

Stage[a]	K (μM^{-1})	ΔH (kCal/mol)	−TΔS (kCal/mol)	Active Fraction (A)	Active Fraction (B)
A + B + B → BA + B	0.1317	−18.756	11.772	0.973	0.965
BA + B → BAB	0.1315	0.952	−7.935		

JIP3^{LZ}. See also Table S1.
[a]A, GST-JIP3^{LZ} dimer; B, KLC2^{TPR}.

The JIP3^{LZ} coiled coil organizes these residues into a continuous KLC2 binding site on one side of the JIP3^{LZ} (Figure 3B). The following describes the KLC2^{TPR}:JIP3^{LZ} interface involving chain A. The side chains of JIP3 residues Ala^{437}, Leu^{438}, Val^{441}, and Leu^{445} (chain D), and Ile^{446} (chain C) form a hydrophobic patch, which is flanked by the hydrophilic residues Glu^{433}, Thr^{434}, and Asp^{450} (chain D) and Lys^{435}, Asn^{439} Lys^{442}, and Asp^{450} (chain C). The JIP3^{LZ} hydrophobic patch is recognized

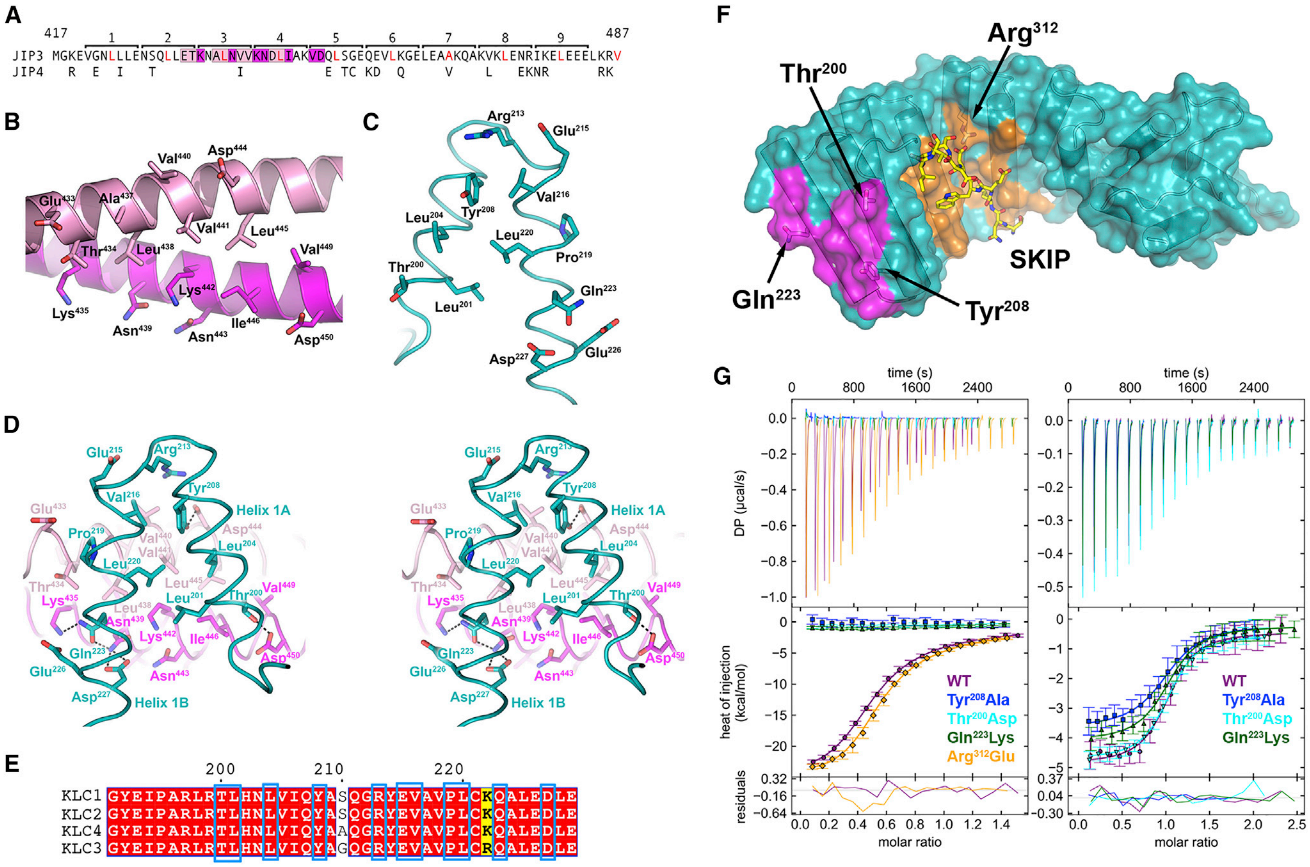

Figure 3. The JIP3^{LZ} Binds to KLC2 TPR1

(A) Amino acid sequence of the murine JIP3^{LZ}. Heptad repeats are numbered and the amino acid in the "d" position is written in red. Residues that bind to KLC2^{TPR} chain A are highlighted in magenta (chain C) and pink (chain D). JIP4 residues not conserved with JIP3 are written beneath.

(B) The KLC2 binding site on the JIP3^{LZ} domain (chain C, magenta; chain D, pink). The side chains of residues that interact with the KLC2^{TPR} domain are shown as sticks (carbon, main chain color scheme; nitrogen, blue; oxygen, red).

(C) The JIP3 binding site on the KLC2 TPR1. The side chains of residues that interact with the JIP3^{LZ} are shown as sticks.

(D) Stereo image of the KLC2^{TPR}:JIP3^{LZ} interface involving KLC2^{TPR} chain A. Putative hydrogen bonds are shown as dashed lines.

(E) Amino acid sequence alignment for murine KLC1-4 over TPR1. Conserved and semi-conserved residues are highlighted in red and yellow, respectively. KLC2 residues that interact with JIP3 and their counterparts in KLC1/3/4 are outlined in blue. Numbering corresponds to KLC2.

(F) The KLC2^{TPR} from the KLC2^{TPR}:JIP3^{LZ} crystal structure showing the JIP3 (magenta) and tryptophan-acidic (orange) binding sites. The tryptophan-acidic peptide from SKIP (sticks; carbon, yellow; nitrogen, blue; oxygen, red) was modeled based on PDB: 3ZFW (Pernigo et al., 2013). TPR1 residues that were mutated in the binding studies in (G) are labeled.

(G) Representative ITC thermograms and isotherms for titration of the Trp-JIP3^{LZ} domain (left panel) or FITC-CSTN-WD2 peptide (right panel) into wild-type and mutant KLC2^{TPR}-myc. Molar ratios for the Trp-JIP3^{LZ} domain correspond to the Trp-JIP3^{LZ} dimer concentration. The error bars in the isotherms show the errors associated with integration of the injection peaks in the corresponding thermograms.

See also Table S2.

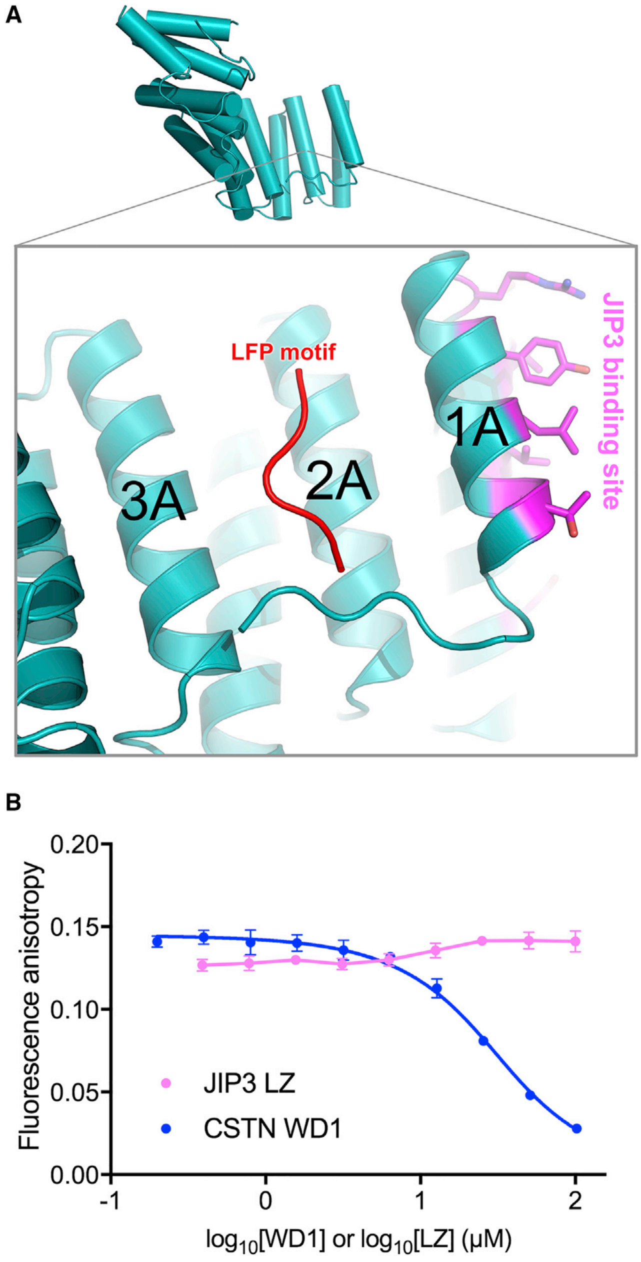

Figure 4. The JIP3^{LZ} Does Not Disrupt LFP Motif Binding to the KLC2^{TPR}

(A) View of the KLC2^{TPR} from the KLC2^{TPR}:JIP3^{LZ} complex. The bound LFP motif (red cartoon) was modeled using PDB: 5FJY (Yip et al., 2016).

(B) Fluorescence anisotropy from the FITC-LFP peptide with a fixed concentration of KLC2^{TPR}-myc (21 μM) and varying concentrations of Trp-JIP3^{LZ} or CSTN-WD1 peptide as indicated. For the Trp-JIP^{LZ} data, concentration values correspond to the total concentration of monomeric Trp-JIP^{LZ} subunits. The calsyntenin-1 data are fitted with a competitive inhibition model (see the STAR Methods). The JIP3 data points are joined by straight lines. Data from a single experiment is shown in each case. Data points and error bars correspond to the mean and SD of triplicate measurements. Where error bars are not visible they are smaller than the marker.

by a number of hydrophobic side chains that line the inside of KLC2 TPR1 (Leu^{201}, Leu^{204}, Tyr^{208}, Val^{216}, and Leu^{220}) (Figure 3C). Around the edge of the hydrophobic core of the KLC2^{TPR}:JIP3^{LZ} interface, the side chains of KLC2 residues Thr^{200} and Tyr^{208} (TPR1 helix A), and Gln^{223} and Asp^{227} (helix B), hydrogen bond to the side chains of JIP3 residues Asp^{450}, Asp^{444}, Lys^{435}, and Asn^{439} (Figure 3D). The interacting residues in the complex are very highly conserved in KLC1 and KLC3-4 and in JIP4 (Figures 3A and 3E), consistent with previous studies (Bowman et al., 2000; Byrd et al., 2001; Nguyen et al., 2005).

To verify that the crystal structure of the KLC2^{TPR}:JIP3^{LZ} complex is the same as in solution, we probed the KLC2^{TPR}:JIP3^{LZ} interface using ITC binding experiments with wild-type and mutant KLC2^{TPR}-myc. (Figure 3F). All three mutations in TPR1 completely abrogated binding of the Trp-JIP3^{LZ}, but had only minor effects on binding of a tryptophan-acidic cargo peptide (Figure 3G; Table S2). In contrast, the Arg^{312}Glu mutant, which is unable to bind to tryptophan-acidic cargoes (Pernigo et al., 2013), retained the ability to bind to the Trp-JIP3^{LZ}. These results validate the crystal structure of the KLC2^{TPR}:JIP3^{LZ} complex and confirm the presence of a cargo binding site on the kinesin-1 molecule, separate from that of tryptophan-acidic cargoes, located on KLC TPR1 (Figure 3F).

JIP3^{LZ} Binding to KLC2^{TPR} Does Not Affect LFP Motif Binding

Kinesin-1 activation by JIP3 requires JIP3^{LZ} binding to KLC^{TPR} (Watt et al., 2015). How does JIP3^{LZ} binding to the KLC^{TPR} relieve KLC regulation of the kinesin-1 molecule? Tryptophan-acidic cargoes activate kinesin-1 by releasing the KLC LFP motif from the KLC^{TPR} (Yip et al., 2016). A previous X-ray crystallographic study at 4 Å resolution suggested that the LFP motif binds to a site on the inner surface of the KLC2^{TPR} located on TPR2, immediately adjacent to TPR1 (Figure 4A) (Yip et al., 2016). We therefore wondered whether JIP3^{LZ} binding to the KLC2^{TPR} allosterically regulates interactions between KLC2^{TPR} and the LFP motif. We investigated this using an *in vitro*, fluorescence anisotropy-based binding competition assay with purified proteins and peptides (Yip et al., 2016). This showed that the Trp-JIP3^{LZ} had no effect on the binding of the KLC2^{TPR}-myc to a fluorescein isothiocyanate (FITC)-labeled LFP motif peptide (Figure 6B). In contrast, a tryptophan-acidic peptide (CSTN-WD1) blocked binding of the fluorescent peptide to the KLC2^{TPR}-myc, with a K_I of 11.3 μM, in line with previous results (Figure 4B) (Yip et al., 2016). This demonstrates that JIP3^{LZ} binding would not release the LFP motif from its binding site on the KLC^{TPR}. Thus, JIP3 activates kinesin-1 by a different mechanism to tryptophan-acidic cargoes.

Evolutionary Analysis Points to a Role for KLC TPR1 beyond JIP3 Binding

We analyzed the conservation of the amino acid residues at the KLC2^{TPR}:JIP3^{LZ} interface using used a recently published dataset containing a significantly enhanced number of non-Bilaterian genomes (Figure 5A; Tables S3 and S4) (Simion et al., 2017). This allowed us to examine the conservation of this binding interface across the full breadth of the animal kingdom. We found that the

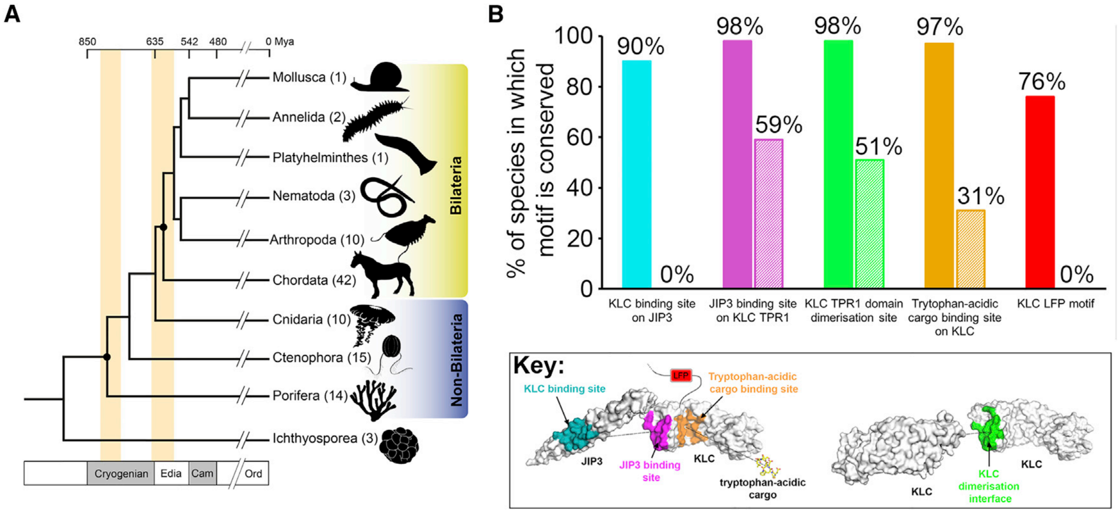

Figure 5. Conservation of the KLC2^{TPR}:JIP3^{LZ} Interface across The Animal Kingdom

(A) Canonical animal species phylogeny complete with the evolutionary timeline. The timing of the emergence of Metazoa and Bilateria in the fossil record is indicated by orange bars. The number of species in our dataset for each phylum is given in parentheses.

(B) Bar graph showing the percentage of species in our datasets containing the indicated KLC or JIP3 motifs. Solid and hatched bars show data for Bilaterian and non-Bilaterian species, respectively. Bars are color-coded by motif as shown in the key.

See also Tables S3 and S4.

KLC2^{TPR} binding site on JIP3 is specific to animals with bilateral body symmetry (Bilateria): the amino acids involved in KLC2^{TPR} binding are totally conserved in almost all Bilaterian JIP3/4 homologs, but not those from any species in the other animal phyla. However, the JIP3^{LZ} binding site on TPR1 is more widely conserved, being totally conserved in essentially all Bilateria, but also in over half of the non-Bilaterian species in our datasets, across all phyla (Figure 5B; Table S4). The two faces of the KLC2^{TPR}:JIP3^{LZ} interface are therefore conserved to very different extents throughout the animal kingdom. Thus, while JIP3 evolved to bind to the KLC^{TPR} at or shortly after the divergence of Bilateria, the cognate region of the KLC^{TPR} evolved much earlier, or evolved multiple times in the absence of JIP3^{LZ} binding. For comparison, the KLC LFP motif was found to be specific to Bilateria, while the tryptophan-acidic cargo binding site was present in 31% of non-Bilaterian species in addition to being fully conserved in essentially all Bilateria (Figure 5B). We conclude that the JIP3^{LZ} binding site on the KLC^{TPR} is the most widely conserved binding interface on KLC mapped to date. This points to additional roles for KLC TPR1 in kinesin-1 function beyond serving as a binding site for the JIP3^{LZ}, perhaps in binding to other, as-yet unknown cargoes, and/or as a regulator of kinesin-1 activity.

TPR1 Forms a Conserved Crystallographic Interface that Resembles JIP3 Binding

Intriguingly, TPR1 forms a similar dimer interface in all crystal structures of the full-length KLC^{TPR} (Figures S2A and S2B) (Nguyen et al., 2017). Notably, we also found the same interface in our previously unpublished, 4-Å crystal structure of the KLC2^{TPR} bound to a tryptophan-acidic peptide derived from calsyntenin-1 (CSTN-WD2) (Figure S2C; Table 1) (Araki et al., 2007). The TPR1:TPR1 packing interaction is unique in being found in every copy of the KLC1^{TPR} and KLC2^{TPR} in all crystal structures (Figure S2D), suggesting that it is independent of crystallographic environment or crystallization conditions (Table S5). The dimer interfaces in PDB: 3NF1, 5FJY, and our KLC2^{TPR}:CSTN-WD2 structure are very similar, with pairwise superposition of these dimers via TPR1 giving root-mean-square deviation (RMSD) values of 0.7–1.0 Å between 81 and 83 structurally equivalent C_α atoms (Figure S2E). The amino acid residues that form the crystallographic TPR1:TPR1 interfaces are totally conserved in essentially all Bilateria and over half of the non-Bilaterian species in our datasets, across all phyla (Figure 5B; Tables S3 and S4). These observations suggest that the TPR1:TPR1 interfaces observed in crystals of the KLC^{TPR} are not simply crystal packing artifacts and may be functionally relevant.

We compared the KLC2^{TPR}:JIP3^{LZ} interface with the crystallographic TPR1:TPR1 packing interfaces observed in other KLC^{TPR} crystal structures (Nguyen et al., 2017; Yip et al., 2016; Zhu et al., 2012). The conformation of TPR1 in complex with the JIP3^{LZ} is essentially identical to that observed in crystals of the unbound, full-length KLC2^{TPR} (PDB: 5OJF; Nguyen et al., 2017), with pairwise RMSD of 0.4–0.6 Å over 41 equivalent C_α atoms (Figure S2F). Remarkably, TPR1 dimerization in PDB: 5OJF is strikingly similar to JIP3^{LZ} binding (Figures 6A–6C), with most of the residues in the JIP3 binding site engaged in the TPR1 dimer interface (Figure 6D). Comparison with the other KLC^{TPR} structures, which possess a slightly different TPR1 conformation, showed similar results, with an even greater overlap between the JIP3 binding site and the TPR1 dimerization interface (Figure 6E). Thus, the KLC^{TPR} possesses the propensity to self-associate via TPR1 in a manner that mimics JIP3^{LZ} domain binding. Since JIP3 binding to KLC TPR1 is required for activation of the kinesin-1 heterotetramer, self-association

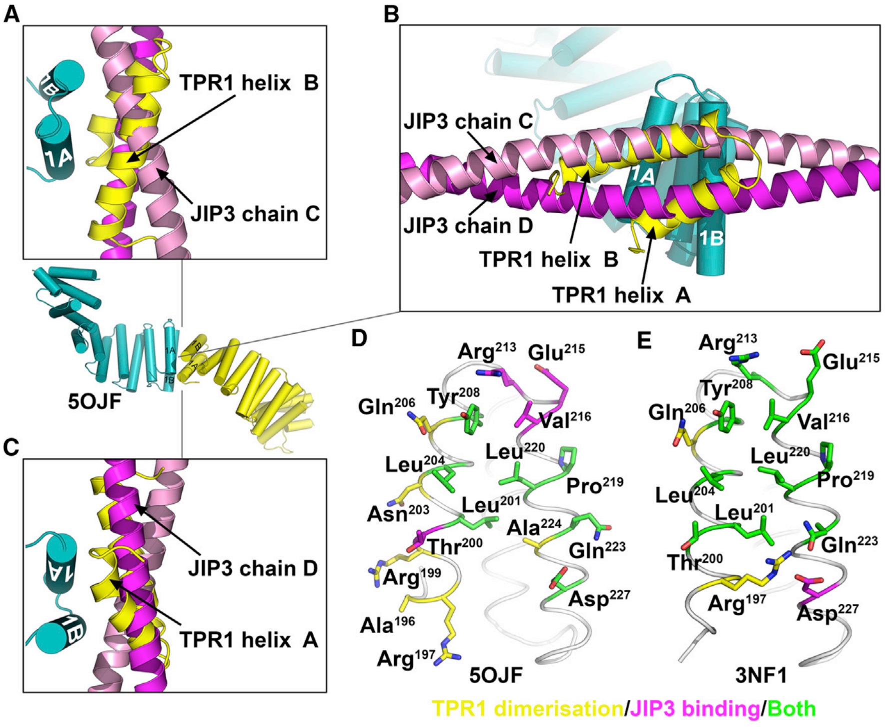

Figure 6. KLC^{TPR} Dimerization via TPR1 Mimics JIP3 Binding

(A–C) Structural comparison of the KLC2^{TPR}:JIP3^{LZ} interface with the crystallographic TPR1:TPR1 dimer interface in crystals of the KLC2^{TPR} (PDB: 5OJF). (A–C) show three views of the PDB: 5OJF dimer interface, with chain A in teal and the dimer mate in yellow. The JIP3^{LZ} (pink and magenta) was positioned onto PDB: 5OJF chain A by superposing KLC2 chain A of the KLC2^{TPR}:JIP3^{LZ} complex onto subunit A of PDB: 5OJF via TPR1.

(D–E) TPR1 from KLC2 (D) and KLC1 (E) shown in gray loop representation (PDB: 5OJF and 3NF1, respectively). Interfacial residues are shown in stick representation with carbon atoms colored according to the interface(s) they participate in.

See also Figure S2.

of the two KLC^{TPR}s inside the kinesin-1 molecule via TPR1 could also play a role in kinesin-1 activation.

DISCUSSION

While most eukaryotes possess a single cytoplasmic dynein that functions in conjunction with a multitude of regulators, eukaryotic genomes typically encode multiple kinesins. Each kinesin contains a conserved motor domain, with variable flanking regions that regulate the activity of the motor domains and mediate binding to effectors, e.g., cargoes (Hirokawa et al., 2009). An emerging theme is that kinesin motor domains are regulated by intramolecular interactions with the flanking non-motor regions (Espeut et al., 2008; Hackney et al., 2009; Hackney and Stock, 2000; Hammond et al., 2010; Kaan et al., 2011; Stock et al., 1999; Talapatra et al., 2015). These interactions are relieved by cargo binding, post-translational modifications, or, in the case of microtubule depolymerizing kinesins, even microtubule ends themselves (Espeut et al., 2008; Friedman and Vale, 1999; Talapatra et al., 2015; Welburn, 2013). In the kinesin-1 heterotetramer, motor domain regulation involves a direct interaction between the KHC tail and motor domains, and an additional, poorly understood role for KLC. Here, we have solved the crystal structure of the KLC2^{TPR} bound to the cognate region of a cargo molecule, the JIP3^{LZ}. In the following we explore the implications of this structure for our molecular understanding of kinesin-1 recruitment and activation by cargo.

Molecular Mechanisms by which ARF6 Regulates Kinesin-1 Recruitment by JIP3/4

JIP3 and JIP4 are molecular adaptors that link kinesin-1 and dynein to a range of other cellular cargoes, expanding the repertoire of cellular cargoes that can be transported by these microtubule motors. The switch between kinesin-1 and dynein-driven motility is controlled by ARF6 (Montagnac et al., 2009). Comparison with the crystal structure of the ARF6:JIP4^{LZ} complex shows that the KLC and ARF6 binding sites on the JIP3/4^{LZ} overlap (Figure 7A), consistent with KLC and ARF6 competing directly for binding (Montagnac et al., 2009). However, only one molecule of ARF6 can bind to the JIP3/4^{LZ} dimer, because active ARF6 is anchored into membranes via its N-terminal myristoyl group (Gillingham and Munro, 2007; Isabet et al., 2009). As a result, the other ARF6/KLC binding site on the JIP3/4^{LZ} would be vacant. Comparison with our KLC2^{TPR}:JIP3^{LZ} structure shows that the vacant binding site would be inaccessible to the KLC2^{TPR} due to its proximity to the membrane (Figure 7B). Thus, one molecule of activated ARF6 blocks KLC^{TPR} binding to both sites on the JIP3/4^{LZ}.

Insights into Kinesin-1 Activation by Cargo

JIP3 binding to the KLC^{TPR} relieves KLC regulation of the kinesin-1 molecule. TPR1 adopts two slightly different conformations in crystal structures of unbound KLC^{TPR}s (Figures 6D and 6E) (Nguyen et al., 2017). Conformational changes in

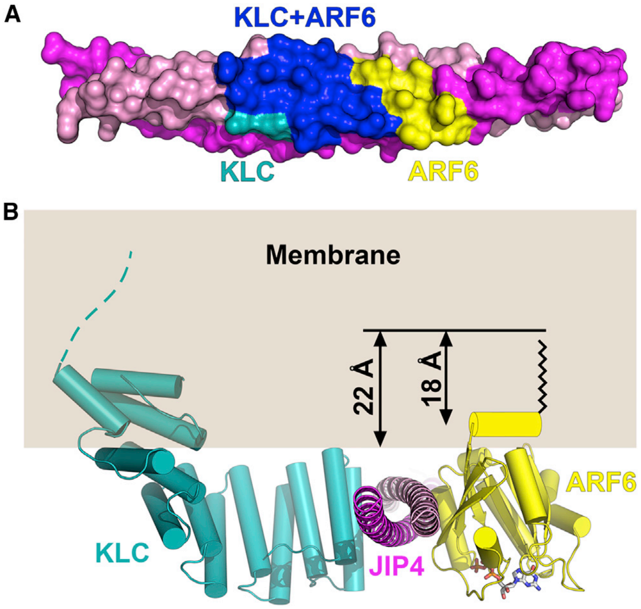

Figure 7. Molecular Mechanisms by which ARF6 Regulates Kinesin-1 Recruitment by JIP3/4

(A) Molecular surface of the JIP4^{LZ} (PDB: 2W83) showing the KLC and ARF6 binding sites (Isabet et al., 2009).

(B) Model of the JIP4^{LZ} bound to the KLC2^{TPR} and active, membrane-associated ARF6. GTP, sticks with carbon/oxygen/nitrogen/phosphorus, gray/red/blue/orange; N-terminal myristoyl group, black zigzag; N-terminal amphipathic helix, yellow cylinder.

TPR1 induced by JIP3 binding could form part of an allosteric mechanism for relieving KLC regulation. However, we found that JIP3 does not disrupt interactions between the KLC LFP motif and the TPR domain (Figure 4B). Thus, JIP3 activates kinesin-1 through a different mechanism to tryptophan-acidic cargoes. Interestingly, we found that the KLC binding site on JIP3 and the KLC LFP motif co-evolved at an early stage of the Bilaterian split (Figure 6B), suggesting a functional relationship between the LFP motif and JIP3 binding to KLC. Structural information on the intact kinesin-1 molecule is scarce, and thus how KLC interacts with KHC in the autoinhibited state is poorly understood. Previous results are consistent with the KLC acidic linker and TPR domain forming interactions with the KHC motor and/or tail domains in the autoinhibited state (Wong and Rice, 2010). Fluorescence lifetime imaging experiments showed that the KLC N- and C-termini are in close spatial proximity inside the kinesin-1 molecule, and that this configuration requires LFP motif binding to the TPR domain (Yip et al., 2016). Thus, the LFP motif would naturally direct the JIP3 binding site on the KLC^{TPR} toward the KHC motor and tail domains in the autoinhibited state (Figure 8A). This points to a role for the KLC LFP motif in scaffolding regulatory interactions between the JIP3 binding site (or adjoining regions of KLC) and the KHC motor domains/tails. Within this picture, cargoes could relieve these regulatory interactions either through binding to KLC TPR1, as for JIP3, or by dissociating the LFP motif from the TPR domain, as with tryptophan-acidic cargoes. Previously, it was proposed that the KLC acidic linker (Figure 1A) is an activator of kinesin-1 motility, and that binding of the LFP motif to the KLC^{TPR} constitutes an autoinhibition mechanism (Yip et al., 2016). Our hypothesis above is entirely compatible with this model, but suggests additional functions for the LFP motif, allowing us to reconcile the activation pathways of different cargoes into a common molecular framework.

TPR1 forms similar crystal packing interfaces in all crystal structures of the KLC^{TPR} (Figure S2) (Nguyen et al., 2017). However, it was unclear whether these TPR1 conformations and the corresponding TPR1:TPR1 interfaces correspond to functionally relevant states of the protein or represent crystal packing artifacts. We have shown that TPR1 harbors the binding site for a cargo, JIP3, and that the JIP3-bound TPR1 conformation is virtually identical to that observed in crystals of the unbound KLC2^{TPR} (PDB: 5OJF) (Figure S2F) (Nguyen et al., 2017). This shows that the TPR1:TPR1 dimer interface in PDB: 5OJF involves a functional TPR1 conformation. TPR1-mediated dimerization mimics JIP3 binding (Figures 6A–6C), and uses an almost identical set of residues that are conserved in KLC homologs from sponges to humans (Figures 6D and 6E; Tables S3 and S4). These observations argue strongly in favor of a functional role for TPR1-mediated dimerization of the KLC^{TPR}s in the kinesin-1 molecule. Since JIP3 binding to TPR1 relieves KLC regulation (Watt et al., 2015), then so should dimerization of the KLC^{TPR}s via TPR1. It thus appears that, following the emergence of LFP motif-mediated KLC regulation in Bilateria, JIP3 evolved to relieve this regulation by co-opting an ancestral TPR1-mediated dimerization mechanism. Purified, recombinant KLC^{TPR}s are monomeric in solution, showing that this interaction is weak (Nguyen et al., 2017). Considering the two KLC^{TPR}s inside the kinesin-1 molecule as confined to a sphere of maximum radius 150 Å (corresponding to a fully extended acidic linker) shows that the KLC^{TPR} concentration inside the kinesin-1 molecule would be at least 100 μM. Nevertheless, we anticipate that the monomer-dimer equilibrium of KLC^{TPR}s in the kinesin-1 molecule would favor the monomer in the autoinhibited state, since dimerization would mask the JIP3 binding site and lead to relief of KLC regulation. However, cargoes that tether the TPR domains together would promote dimerization via TPR1, leading to relief of KLC regulation. Indeed, KLC cargoes are usually dimeric or possess two KLC binding sites. This would allow cargoes binding to different regions of the KLC^{TPR} to relieve KLC regulation by the same mechanism. For example, kinesin-1 activation by JIP1 (which is structurally unrelated to JIP3/4) follows a similar scheme to JIP3 despite engaging the KLC^{TPR} in a completely different manner (Blasius et al., 2007; Fu and Holzbaur, 2013; Sun et al., 2011; Verhey et al., 2001; Watt et al., 2015; Zhu et al., 2012). Interestingly, JIP1 and JIP3 promote each other's transport inside cells, which requires binding of both cargoes to the KLC^{TPR} (Hammond et al., 2008; Sun et al., 2017). Unrelated cargoes could facilitate each other's transport by cooperating with each other to promote KLC^{TPR} dimerization.

An important functional requirement of molecular motors such as kinesin-1 is the ability to transport multiple, unrelated cellular cargoes. Indeed, the KLC^{TPR} possesses separate binding sites for multiple cargoes, yet how the kinesin-1 molecule integrates these seemingly unrelated molecular recognition

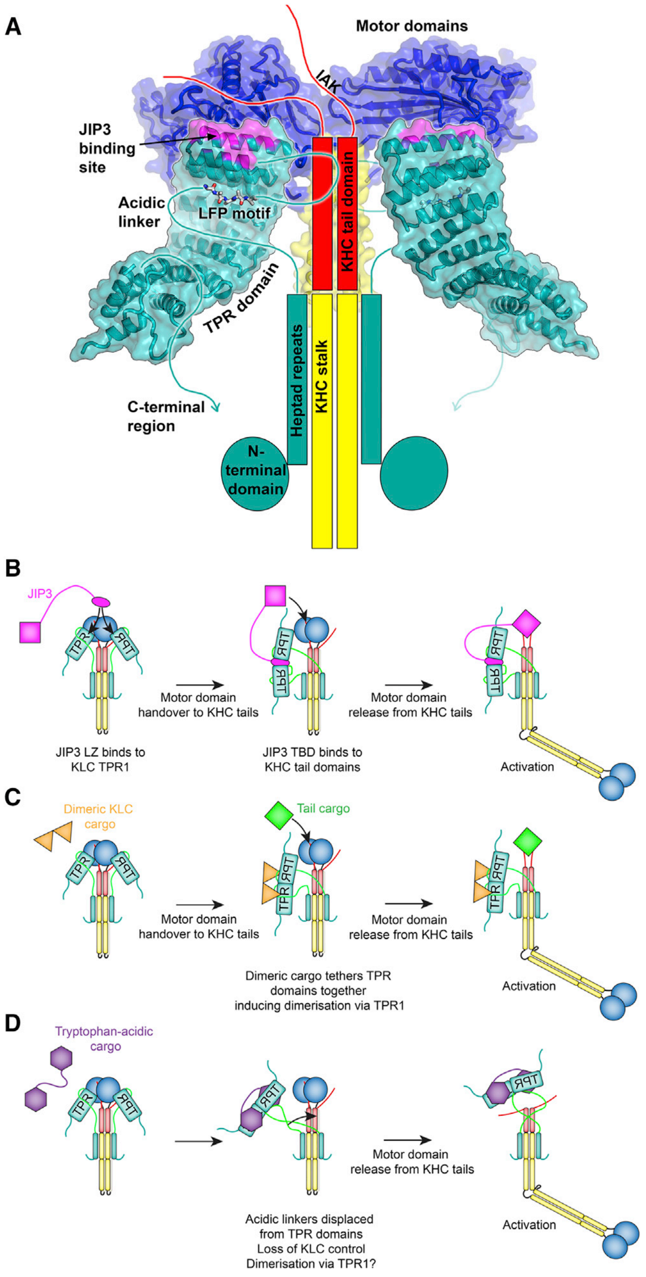

Figure 8. A Unified Framework for Kinesin-1 Activation by Different Cargoes

(A) Model for the autoinhibited state of the kinesin-1 heterotetramer, showing how binding of the KLC LFP motif to the TPR domain would steer the JIP3 binding site on KLC toward the KHC motor domains and tails.

(B) Proposed pathway for kinesin-1 activation by JIP3 (LZ domain, oval; tail-binding region, square).

(C) Proposed pathway for kinesin-1 activation by a general dimeric cargo that induces TPR dimerization via TPR1.

(D) Proposed pathway for kinesin-1 activation by a tryptophan-acidic cargo (tryptophan-acidic motif, hexagon). This has been conceptually broken down into two steps to show relief of KLC and KHC tail regulation of the motor domains, but both steps could occur simultaneously (Yip et al., 2016).

events into a common activation pathway is unknown. Based on our observations, we suggest the following model to explain this (Figures 8B–8D). Binding of the LFP motif to the KLC^{TPR} promotes regulatory interactions between KLC and KHC that involve KLC TPR1 or nearby regions. Cargoes can therefore relieve KLC regulation by one of two mechanisms. The first is crosslinking the KLC^{TPR}s together via their TPR1s. This could occur via direct binding to TPR1, as seen for JIP3 (Figure 8B), or by promoting dimerization of the TPR domains via TPR1 (Figure 8C). The second mechanism for relieving KLC regulation involves "pulling the rug out from underneath it," i.e., dissociating the KLC LFP motif from the TPR domain, as seen for tryptophan-acidic cargoes (Figure 8D). Whichever way KLC regulation is disabled, it results in an intermediate that resembles the KHC homodimer, in which the motor domains are autoinhibited by the KHC tails alone. Binding of other factors to the KHC tail is then required to activate motility. This could be a cargo molecule (Figures 8B and 8C) (Blasius et al., 2007; Fu and Holzbaur, 2013; Sun et al., 2011; Watt et al., 2015), or, as suggested previously, the KLC acidic linker (Figure 8D) (Yip et al., 2016), although recent work shows that robust activation of kinesin-1 by tryptophan-acidic cargoes requires cargo binding to the KHC tails (Sanger et al., 2017). Detailed information on the structure of the intact kinesin-1 molecule and further mechanistic studies will be needed to investigate these issues further.

In summary, this work expands our knowledge of how cargoes recruit kinesin-1, how this is regulated by factors such as ARF6, and suggests how multiple, unrelated cargoes binding to different sites on the KLC TPR domain can activate kinesin-1 through related mechanisms.

STAR★METHODS

Detailed methods are provided in the online version of this paper and include the following:

- KEY RESOURCES TABLE
- CONTACT FOR REAGENT AND RESOURCE SHARING
- EXPERIMENTAL MODEL AND SUBJECT DETAILS
- METHOD DETAILS
  - Construction of Expression Vectors
  - Protein Production
  - Protein Purification
  - Crystal Structure Determination
  - Isothermal Titration Calorimetry
  - CD Spectroscopy
  - SEC-MALLS Experiments
  - Binding Competition Experiments
  - Bioinformatic Sequence Analyses
  - Structural Analyses
  - Crystal Packing Analysis
- QUANTIFICATION AND STATISTICAL ANALYSIS
- DATA AND SOFTWARE AVAILABILITY

SUPPLEMENTAL INFORMATION

Supplemental Information includes two figures and five tables and can be found with this article online at https://doi.org/10.1016/j.str.2018.07.011.

ACKNOWLEDGMENTS

We gratefully acknowledge assistance from Dr. Iain Manfield, Dr. Chi Trinh, Dr. G. Nasir Khan, and Dr. Amy Barker (University of Leeds), and Dr. Nicola O'Reilly (Peptide Synthesis Laboratory, Francis Crick Institute). We also thank Dr. Roberto Steiner (King's College, University of London) and Dr. Mark Dodding (University of Bristol) for helpful discussions. This work was made possible by grant 094232MA 094232/Z/10/Z from The Wellcome Trust. S.J.H. was supported by a BBSRC White Rose Doctoral Training Fellowship. M.W. is supported by the Francis Crick Institute, which receives its core funding from Cancer Research UK (FC001209), the UK Medical Research Council (FC001209), and the Wellcome Trust (FC001209). J.J.B.C. was part-funded by a Cancer Research UK post-doctoral fellowship from the London Research Institute (Way lab), start-up funding from the University of Leeds, Royal Society Research Grant RG150205, and a Wellcome Trust Seed Award (204576/Z/16/Z).

AUTHOR CONTRIBUTIONS

J.J.B.C. and M.W. conceived the study. J.J.B.C. and S.J.H. solved the crystal structures. J.J.B.C. performed the binding experiments. M.T. performed SEC-MALLS experiments. P.M. and M.J.O'C. performed the molecular evolutionary analyses. J.J.B.C. wrote the paper with contributions from all authors. All authors read and commented on the final version of the manuscript.

DECLARATION OF INTERESTS

The authors declare that they have no competing financial interests.

Received: February 16, 2018
Revised: June 3, 2018
Accepted: July 25, 2018
Published: September 6, 2018

REFERENCES

Adams, P.D., Afonine, P.V., Bunkoczi, G., Chen, V.B., Davis, I.W., Echols, N., Headd, J.J., Hung, L.W., Kapral, G.J., Grosse-Kunstleve, R.W., et al. (2010). PHENIX: a comprehensive Python-based system for macromolecular structure solution. Acta Crystallogr. D Biol. Crystallogr. *66*, 213–221.

Altenhoff, A.M., Skunca, N., Glover, N., Train, C.M., Sueki, A., Pilizota, I., Gori, K., Tomiczek, B., Muller, S., Redestig, H., et al. (2015). The OMA orthology database in 2015: function predictions, better plant support, synteny view and other improvements. Nucleic Acids Res. *43*, D240–D249.

Altschul, S.F., Madden, T.L., Schaffer, A.A., Zhang, J., Zhang, Z., Miller, W., and Lipman, D.J. (1997). Gapped BLAST and PSI-BLAST: a new generation of protein database search programs. Nucleic Acids Res. *25*, 3389–3402.

Araki, Y., Kawano, T., Taru, H., Saito, Y., Wada, S., Miyamoto, K., Kobayashi, H., Ishikawa, H.O., Ohsugi, Y., Yamamoto, T., et al. (2007). The novel cargo Alcadein induces vesicle association of kinesin-1 motor components and activates axonal transport. EMBO J. *26*, 1475–1486.

Blasius, T.L., Cai, D., Jih, G.T., Toret, C.P., and Verhey, K.J. (2007). Two binding partners cooperate to activate the molecular motor Kinesin-1. J. Cell Biol. *176*, 11–17.

Boeda, B., Briggs, D.C., Higgins, T., Garvalov, B.K., Fadden, A.J., McDonald, N.Q., and Way, M. (2007). Tes, a specific Mena interacting partner, breaks the rules for EVH1 binding. Mol. Cell *28*, 1071–1082.

Bowman, A.B., Kamal, A., Ritchings, B.W., Philp, A.V., McGrail, M., Gindhart, J.G., and Goldstein, L.S. (2000). Kinesin-dependent axonal transport is mediated by the sunday driver (SYD) protein. Cell *103*, 583–594.

Byrd, D.T., Kawasaki, M., Walcoff, M., Hisamoto, N., Matsumoto, K., and Jin, Y. (2001). UNC-16, a JNK-signaling scaffold protein, regulates vesicle transport in *C. elegans*. Neuron *32*, 787–800.

Cai, D., Hoppe, A.D., Swanson, J.A., and Verhey, K.J. (2007). Kinesin-1 structural organization and conformational changes revealed by FRET stoichiometry in live cells. J. Cell Biol. *176*, 51–63.

Cavalli, V., Kujala, P., Klumperman, J., and Goldstein, L.S. (2005). Sunday Driver links axonal transport to damage signaling. J. Cell Biol. *168*, 775–787.

Chen, V.B., Arendall, W.B., 3rd, Headd, J.J., Keedy, D.A., Immormino, R.M., Kapral, G.J., Murray, L.W., Richardson, J.S., and Richardson, D.C. (2010). MolProbity: all-atom structure validation for macromolecular crystallography. Acta Crystallogr. D Biol. Crystallogr. *66*, 12–21.

Coy, D.L., Hancock, W.O., Wagenbach, M., and Howard, J. (1999). Kinesin's tail domain is an inhibitory regulator of the motor domain. Nat. Cell. Biol. *1*, 288–292.

Diefenbach, R.J., Diefenbach, E., Douglas, M.W., and Cunningham, A.L. (2002). The heavy chain of conventional kinesin interacts with the SNARE proteins SNAP25 and SNAP23. Biochemistry *41*, 14906–14915.

Dodding, M.P., Mitter, R., Humphries, A.C., and Way, M. (2011). A kinesin-1 binding motif in vaccinia virus that is widespread throughout the human genome. EMBO J. *30*, 4523–4538.

Dodding, M.P., and Way, M. (2011). Coupling viruses to dynein and kinesin-1. EMBO J. *30*, 3527–3539.

Edwards, S.L., Yu, S.C., Hoover, C.M., Phillips, B.C., Richmond, J.E., and Miller, K.G. (2013). An organelle gatekeeper function for *Caenorhabditis elegans* UNC-16 (JIP3) at the axon initial segment. Genetics *194*, 143–161.

Emsley, P., Lohkamp, B., Scott, W.G., and Cowtan, K. (2010). Features and development of Coot. Acta Crystallogr. D Biol. Crystallogr. *66*, 486–501.

Espeut, J., Gaussen, A., Bieling, P., Morin, V., Prieto, S., Fesquet, D., Surrey, T., and Abrieu, A. (2008). Phosphorylation relieves autoinhibition of the kinetochore motor Cenp-E. Mol. Cell *29*, 637–643.

Friedman, D.S., and Vale, R.D. (1999). Single-molecule analysis of kinesin motility reveals regulation by the cargo-binding tail domain. Nat. Cell. Biol. *1*, 293–297.

Frishman, D., and Argos, P. (1995). Knowledge-based protein secondary structure assignment. Proteins *23*, 566–579.

Fu, M.M., and Holzbaur, E.L. (2013). JIP1 regulates the directionality of APP axonal transport by coordinating kinesin and dynein motors. J. Cell Biol. *202*, 495–508.

Gauger, A.K., and Goldstein, L.S. (1993). The *Drosophila* kinesin light chain. Primary structure and interaction with kinesin heavy chain. J. Biol. Chem. *268*, 13657–13666.

Gertz, E.M., Yu, Y.K., Agarwala, R., Schaffer, A.A., and Altschul, S.F. (2006). Composition-based statistics and translated nucleotide searches: improving the TBLASTN module of BLAST. BMC Biol. *4*, 41.

Gillingham, A.K., and Munro, S. (2007). The small G proteins of the Arf family and their regulators. Annu. Rev. Cell Dev. Biol. *23*, 579–611.

Gindhart, J.G., Chen, J., Faulkner, M., Gandhi, R., Doerner, K., Wisniewski, T., and Nandlestadt, A. (2003). The kinesin-associated protein UNC-76 is required for axonal transport in the *Drosophila* nervous system. Mol. Biol. Cell *14*, 3356–3365.

Gindhart, J.G., Jr., Desai, C.J., Beushausen, S., Zinn, K., and Goldstein, L.S. (1998). Kinesin light chains are essential for axonal transport in *Drosophila*. J. Cell Biol. *141*, 443–454.

Glater, E.E., Megeath, L.J., Stowers, R.S., and Schwarz, T.L. (2006). Axonal transport of mitochondria requires milton to recruit kinesin heavy chain and is light chain independent. J. Cell Biol. *173*, 545–557.

Hackney, D.D., Baek, N., and Snyder, A.C. (2009). Half-site inhibition of dimeric kinesin head domains by monomeric tail domains. Biochemistry *48*, 3448–3456.

Hackney, D.D., Levitt, J.D., and Suhan, J. (1992). Kinesin undergoes a 9 S to 6 S conformational transition. J. Biol. Chem. *267*, 8696–8701.

Hackney, D.D., and Stock, M.F. (2000). Kinesin's IAK tail domain inhibits initial microtubule-stimulated ADP release. Nat. Cell. Biol. *2*, 257–260.

Hammond, J.W., Blasius, T.L., Soppina, V., Cai, D., and Verhey, K.J. (2010). Autoinhibition of the kinesin-2 motor KIF17 via dual intramolecular mechanisms. J. Cell Biol. *189*, 1013–1025.

Hammond, J.W., Griffin, K., Jih, G.T., Stuckey, J., and Verhey, K.J. (2008). Co-operative versus independent transport of different cargoes by Kinesin-1. Traffic *9*, 725–741.

Hirokawa, N., Noda, Y., Tanaka, Y., and Niwa, S. (2009). Kinesin superfamily motor proteins and intracellular transport. Nat. Rev. Mol. Cell Biol. *10*, 682–696.

Hristova, K., Wimley, W.C., Mishra, V.K., Anantharamiah, G.M., Segrest, J.P., and White, S.H. (1999). An amphipathic alpha-helix at a membrane interface: a structural study using a novel X-ray diffraction method. J. Mol. Biol. *290*, 99–117.

Huang, S.H., Duan, S., Sun, T., Wang, J., Zhao, L., Geng, Z., Yan, J., Sun, H.J., and Chen, Z.Y. (2011). JIP3 mediates TrkB axonal anterograde transport and enhances BDNF signaling by directly bridging TrkB with kinesin-1. J. Neurosci. *31*, 10602–10614.

Isabet, T., Montagnac, G., Regazzoni, K., Raynal, B., El Khadali, F., England, P., Franco, M., Chavrier, P., Houdusse, A., and Menetrey, J. (2009). The structural basis of Arf effector specificity: the crystal structure of ARF6 in a complex with JIP4. EMBO J. *28*, 2835–2845.

Johnson, C.S., Buster, D., and Scholey, J.M. (1990). Light chains of sea urchin kinesin identified by immunoadsorption. Cell. Motil. Cytoskeleton *16*, 204–213.

Junco, A., Bhullar, B., Tarnasky, H.A., and van der Hoorn, F.A. (2001). Kinesin light-chain KLC3 expression in testis is restricted to spermatids. Biol. Reprod. *64*, 1320–1330.

Kaan, H.Y., Hackney, D.D., and Kozielski, F. (2011). The structure of the kinesin-1 motor-tail complex reveals the mechanism of autoinhibition. Science *333*, 883–885.

Kabsch, W. (2010). XDS. Acta Crystallogr. D Biol. Crystallogr. *66*, 125–132.

Kawano, T., Araseki, M., Araki, Y., Kinjo, M., Yamamoto, T., and Suzuki, T. (2012). A small peptide sequence is sufficient for initiating kinesin-1 activation through part of TPR region of KLC1. Traffic *13*, 834–848.

Kelkar, N., Gupta, S., Dickens, M., and Davis, R.J. (2000). Interaction of a mitogen-activated protein kinase signaling module with the neuronal protein JIP3. Mol. Cell Biol. *20*, 1030–1043.

Kelkar, N., Standen, C.L., and Davis, R.J. (2005). Role of the JIP4 scaffold protein in the regulation of mitogen-activated protein kinase signaling pathways. Mol. Cell Biol. *25*, 2733–2743.

Keller, S., Vargas, C., Zhao, H., Piszczek, G., Brautigam, C.A., and Schuck, P. (2012). High-precision isothermal titration calorimetry with automated peak-shape analysis. Anal. Chem. *84*, 5066–5073.

Krissinel, E., and Henrick, K. (2004). Secondary-structure matching (SSM), a new tool for fast protein structure alignment in three dimensions. Acta Crystallogr. D Biol. Crystallogr. *60*, 2256–2268.

Marchesin, V., Castro-Castro, A., Lodillinsky, C., Castagnino, A., Cyrta, J., Bonsang-Kitzis, H., Fuhrmann, L., Irondelle, M., Infante, E., Montagnac, G., et al. (2015). ARF6-JIP3/4 regulate endosomal tubules for MT1-MMP exocytosis in cancer invasion. J. Cell Biol. *211*, 339–358.

McCoy, A.J., Grosse-Kunstleve, R.W., Adams, P.D., Winn, M.D., Storoni, L.C., and Read, R.J. (2007). Phaser crystallographic software. J. Appl. Crystallogr. *40*, 658–674.

Montagnac, G., Sibarita, J.B., Loubery, S., Daviet, L., Romao, M., Raposo, G., and Chavrier, P. (2009). ARF6 Interacts with JIP4 to control a motor switch mechanism regulating endosome traffic in cytokinesis. Curr. Biol. *19*, 184–195.

Morfini, G.A., You, Y.M., Pollema, S.L., Kaminska, A., Liu, K., Yoshioka, K., Bjorkblom, B., Coffey, E.T., Bagnato, C., Han, D., et al. (2009). Pathogenic huntingtin inhibits fast axonal transport by activating JNK3 and phosphorylating kinesin. Nat. Neurosci. *12*, 864–871.

Nguyen, Q., Lee, C.M., Le, A., and Reddy, E.P. (2005). JLP associates with kinesin light chain 1 through a novel leucine zipper-like domain. J. Biol. Chem. *280*, 30185–30191.

Nguyen, T.Q., Chenon, M., Vilela, F., Velours, C., Aumont-Nicaise, M., Andreani, J., Varela, P.F., Llinas, P., and Menetrey, J. (2017). Structural plasticity of the N-terminal capping helix of the TPR domain of kinesin light chain. PLoS One *12*, e0186354.

Pernigo, S., Lamprecht, A., Steiner, R.A., and Dodding, M.P. (2013). Structural basis for kinesin-1:cargo recognition. Science *340*, 356–359.

Rahman, A., Friedman, D.S., and Goldstein, L.S. (1998). Two kinesin light chain genes in mice. Identification and characterization of the encoded proteins. J. Biol. Chem. *273*, 15395–15403.

Rahman, A., Kamal, A., Roberts, E.A., and Goldstein, L.S. (1999). Defective kinesin heavy chain behavior in mouse kinesin light chain mutants. J. Cell Biol. *146*, 1277–1288.

Randall, T.S., Yip, Y.Y., Wallock-Richards, D.J., Pfisterer, K., Sanger, A., Ficek, W., Steiner, R.A., Beavil, A.J., Parsons, M., and Dodding, M.P. (2017). A small-molecule activator of kinesin-1 drives remodeling of the microtubule network. Proc. Natl. Acad. Sci. USA *114*, 13738–13743.

Sanger, A., Yip, Y.Y., Randall, T.S., Pernigo, S., Steiner, R.A., and Dodding, M.P. (2017). SKIP controls lysosome positioning using a composite kinesin-1 heavy and light chain-binding domain. J. Cell. Sci. *130*, 1637–1651.

Schulman, V.K., Folker, E.S., Rosen, J.N., and Baylies, M.K. (2014). Syd/JIP3 and JNK signaling are required for myonuclear positioning and muscle function. PLoS Genet. *10*, e1004880.

Seiler, S., Kirchner, J., Horn, C., Kallipolitou, A., Woehlke, G., and Schliwa, M. (2000). Cargo binding and regulatory sites in the tail of fungal conventional kinesin. Nat. Cell. Biol. *2*, 333–338.

Simion, P., Philippe, H., Baurain, D., Jager, M., Richter, D.J., Di Franco, A., Roure, B., Satoh, N., Queinnec, E., Ereskovsky, A., et al. (2017). A Large and consistent phylogenomic dataset supports sponges as the sister group to all other animals. Curr. Biol. *27*, 958–967.

Sreerama, N., Venyaminov, S.Y., and Woody, R.W. (1999). Estimation of the number of alpha-helical and beta-strand segments in proteins using circular dichroism spectroscopy. Protein Sci. *8*, 370–380.

Stock, M.F., Guerrero, J., Cobb, B., Eggers, C.T., Huang, T.G., Li, X., and Hackney, D.D. (1999). Formation of the compact confomer of kinesin requires a COOH-terminal heavy chain domain and inhibits microtubule-stimulated ATPase activity. J. Biol. Chem. *274*, 14617–14623.

Su, Q., Cai, Q., Gerwin, C., Smith, C.L., and Sheng, Z.H. (2004). Syntabulin is a microtubule-associated protein implicated in syntaxin transport in neurons. Nat. Cell. Biol. *6*, 941–953.

Sun, F., Zhu, C., Dixit, R., and Cavalli, V. (2011). Sunday Driver/JIP3 binds kinesin heavy chain directly and enhances its motility. EMBO J. *30*, 3416–3429.

Sun, T., Li, Y., Li, T., Ma, H., Guo, Y., Jiang, X., Hou, M., Huang, S., and Chen, Z. (2017). JIP1 and JIP3 cooperate to mediate TrkB anterograde axonal transport by activating kinesin-1. Cell Mol. Life Sci. *74*, 4027–4044.

Talapatra, S.K., Harker, B., and Welburn, J.P. (2015). The C-terminal region of the motor protein MCAK controls its structure and activity through a conformational switch. Elife *4*, https://doi.org/10.7554/eLife.06421.001.

Vale, R.D. (2003). The molecular motor toolbox for intracellular transport. Cell *112*, 467–480.

van Stokkum, I.H., Spoelder, H.J., Bloemendal, M., van Grondelle, R., and Groen, F.C. (1990). Estimation of protein secondary structure and error analysis from circular dichroism spectra. Anal. Biochem. *191*, 110–118.

Verhey, K.J., Lizotte, D.L., Abramson, T., Barenboim, L., Schnapp, B.J., and Rapoport, T.A. (1998). Light chain-dependent regulation of Kinesin's interaction with microtubules. J. Cell Biol. *143*, 1053–1066.

Verhey, K.J., Meyer, D., Deehan, R., Blenis, J., Schnapp, B.J., Rapoport, T.A., and Margolis, B. (2001). Cargo of kinesin identified as JIP scaffolding proteins and associated signaling molecules. J. Cell Biol. *152*, 959–970.

Watt, D., Dixit, R., and Cavalli, V. (2015). JIP3 activates kinesin-1 motility to promote axon elongation. J. Biol. Chem. *290*, 15512–15525.

Welburn, J.P. (2013). The molecular basis for kinesin functional specificity during mitosis. Cytoskeleton (Hoboken) *70*, 476–493.

Whitmore, L., and Wallace, B.A. (2008). Protein secondary structure analyses from circular dichroism spectroscopy: methods and reference databases. Biopolymers *89*, 392–400.

Winn, M.D., Ballard, C.C., Cowtan, K.D., Dodson, E.J., Emsley, P., Evans, P.R., Keegan, R.M., Krissinel, E.B., Leslie, A.G., McCoy, A., et al. (2011).

Overview of the CCP4 suite and current developments. Acta Crystallogr. D Biol. Crystallogr. *67*, 235–242.

Wong, Y.L., and Rice, S.E. (2010). Kinesin's light chains inhibit the head- and microtubule-binding activity of its tail. Proc. Natl. Acad. Sci. USA *107*, 11781–11786.

Yip, Y.Y., Pernigo, S., Sanger, A., Xu, M., Parsons, M., Steiner, R.A., and Dodding, M.P. (2016). The light chains of kinesin-1 are autoinhibited. Proc. Natl. Acad. Sci. USA *113*, 2418–2423.

Zeytuni, N., and Zarivach, R. (2012). Structural and functional discussion of the tetra-trico-peptide repeat, a protein interaction module. Structure *20*, 397–405.

Zhao, H., Piszczek, G., and Schuck, P. (2015). SEDPHAT – a platform for global ITC analysis and global multi-method analysis of molecular interactions. Methods *76*, 137–148.

Zhou, N.E., Kay, C.M., and Hodges, R.S. (1994). The net energetic contribution of interhelical electrostatic attractions to coiled-coil stability. Protein Eng. *7*, 1365–1372.

Zhu, H., Lee, H.Y., Tong, Y., Hong, B.S., Kim, K.P., Shen, Y., Lim, K.J., Mackenzie, F., Tempel, W., and Park, H.W. (2012). Crystal structures of the tetratricopeptide repeat domains of kinesin light chains: insight into cargo recognition mechanisms. PLoS One *7*, e33943.

STAR★METHODS

KEY RESOURCES TABLE

REAGENT or RESOURCE	SOURCE	IDENTIFIER
Bacterial and Virus Strains		
BL21 (DE3) Rosetta	Merck Millipore	Cat#70954
Chemicals, Peptides, and Recombinant Proteins		
FITC-labeled CSTN(WD2) peptide (FITC-TRQLEWDDSTL-COOH)	Biomatik	N/A
FITC-labeled KLC2 LFP motif peptide (FITC-DSLDDLFPNEDEQS-COOH)	Biomatik	N/A
N-terminally acetylated CSTN-WD1 peptide (Ac-GKENEMDWDDSALTITVN-COOH)	Peptide Synthesis Laboratory, Francis Crick Institute, UK	N/A
N-terminally acetylated CSTN-WD2 peptide (Ac-NATRQLEWDDSTLSY-COOH)	Peptide Synthesis Laboratory, Francis Crick Institute, UK	N/A
$KLC2^{TPR}$ protein	This study	N/A
$KLC2^{TPR}$-myc protein	This study	N/A
$KLC2^{TPR}$-myc Tyr^{208}Ala mutant protein	This study	N/A
$KLC2^{TPR}$-myc Thr^{200}Asp mutant protein	This study	N/A
$KLC2^{TPR}$-myc Gln^{223}Lys mutant protein	This study	N/A
GST3C-$JIP3^{LZ}$ protein	This study	N/A
$JIP3^{LZ}$ protein	This study	N/A
Trp-$JIP3^{LZ}$ protein	This study	N/A
cOmplete EDTA-free protease inhibitor cocktail	Roche	Cat#4693116001
His-trap HP column (1 mL)	GE Life Sciences	Cat#17524701
Glutathione sepharose 4B resin	GE Life Sciences	Cat#17075601
Superdex 75 16 60	GE Life Sciences	Cat#28989333
Superdex 200 16 60	GE Life Sciences	Cat#28989335
HiTrap Q XL column	GE Life Sciences	Cat#17515801
50% (w/v) PEG 3350 solution	Rigaku	Cat#1008054
1M sodium thiocyante solution	Rigaku	Cat#1008268
JCSG Core screens I-IV	Qiagen	Cat#130924-7
Wizard Classic 3 & 4 HT96 screen	Molecular Dimensions	Cat#MD15-W34-B
Deposited Data		
Atomic coordinates and structure factors	This study	PDB: 6EJN
Atomic coordinates and structure factors	This study	PDB: 6F9I
Bioinformatics sequence dataset 1	OMA Orthology database (Altenhoff et al., 2015)	https://omabrowser.org/oma/home/
Bioinformatics sequence dataset 2	Simion et al., 2017	N/A
Atomic coordinates	Zhu et al., 2012	PDB: 3CEQ
Atomic coordinates	Zhu et al., 2012	PDB: 3NF1
Atomic coordinates	Isabet et al., 2009	PDB: 2W83
Atomic coordinates	Pernigo et al., 2013	PDB:3ZFW
Atomic coordinates	Yip et al., 2016	PDB: 5FJY
Atomic coordinates	Nguyen et al., 2017	PDB: 5OJF
Recombinant DNA		
pMW GST-3C vector	Boeda et al., 2007	N/A
pMW His-Sumo vector	This study	N/A
murine KLC2 cDNA	Dodding et al., 2011	N/A
murine JIP3 isoform A cDNA	GenomeCUBE	Cat#IRAVp968C03156D

(*Continued on next page*)

Continued

REAGENT or RESOURCE	SOURCE	IDENTIFIER
Software and Algorithms		
XDS	Kabsch, 2010	http://xds.mpimf-heidelberg.mpg.de/
CCP4 program suite	Winn et al., 2011	http://www.ccp4.ac.uk
COOT	Emsley et al., 2010	https://www2.mrc-lmb.cam.ac.uk/personal/pemsley/coot/
BUSTER 2.10.3	Global Phasing Ltd.	https://www.globalphasing.com
MOLPROBITY	Chen et al., 2010	http://molprobity.biochem.duke.edu
PHENIX	Adams et al., 2010	https://www.phenix-online.org/documentation/reference/refinement.html
NITPIC	Keller et al., 2012	http://biophysics.swmed.edu/MBR/software.html
SEDPHAT	Zhao et al., 2015	http://www.analyticalultracentrifugation.com/sedphat/
GUSSI	University of Texas Southwestern Medical Center	http://biophysics.swmed.edu/MBR/software.html
Prism 5.0	GraphPad	https://www.graphpad.com/scientific-software/prism/
BLASTp	Altschul et al., 1997	https://blast.ncbi.nlm.nih.gov/Blast.cgi?CMD=Web&PAGE_TYPE=BlastDocs&DOC_TYPE=Download
TBLASTN	Gertz et al., 2006	https://blast.ncbi.nlm.nih.gov/Blast.cgi?CMD=Web&PAGE_TYPE=BlastDocs&DOC_TYPE=Download
PYMOL	Schrodinger LLC	http://www.pymol.org
DICHROWEB	Whitmore and Wallace, 2008	http://dichroweb.cryst.bbk.ac.uk/html/references.shtml

CONTACT FOR REAGENT AND RESOURCE SHARING

Further information and requests for resources and reagents should be directed to and will be fulfilled by the Lead Contact, Joseph Cockburn (j.j.b.cockburn@leeds.ac.uk)

EXPERIMENTAL MODEL AND SUBJECT DETAILS

We used *E.coli* Rosetta (DE3) cells for production of all recombinant proteins used in this study. The cells were cultured using standard practices in LB media.

METHOD DETAILS

Construction of Expression Vectors

Plasmids pMW his-SUMO and pMW GST3C (Boeda et al., 2007) are bacterial expression vectors from the Way Lab at the Francis Crick Institute, London, UK. These vectors contain a T7 promoter and encode an N-terminal hexahistidine-SUMO tag, or a GST tag followed and a 3C protease cleavage site, respectively.

The KLC2^{TPR} construct used in this work comprised murine KLC2 residues 191-480, which is similar to those used in previous studies (Nguyen et al., 2017; Zhu et al., 2012) but with a slightly longer N-terminal region to include residues 191-194, which are very highly conserved in Bilaterian KLCs. To construct the pMW-His$_6$-SUMO-KLC2^{TPR} vector, the sequence encoding KLC2^{TPR} (residues 191-480) was amplified by PCR from a murine template (Dodding et al., 2011) and cloned into the NotI/EcoRI sites of pMW his-SUMO. The His$_6$-SUMO- KLC2^{TPR}-myc vectors were constructed likewise, but using a reverse primer sequence encoding the KLC^{TPR} C-terminal region up to residue 480, a BglII site, and an in-frame myc-tag sequence, thus fusing the amino acid sequence RSEQKLISEEDL to the KLC2^{TPR} C-terminus. KLC2^{TPR}-myc mutants (Thr^{200}Asp, Tyr^{208}Ala, Gln^{223}Lys and Arg^{312}Glu) were generated by overlap PCR.

The JIP3^{LZ} domain construct used in this work (residues 417-487 of murine JIP3 isoform A) is equivalent to the JIP4^{LZ} construct that was previously co-crystallised in complex with ARF6 (Isabet et al., 2009). To construct the pMW-GST3C-JIP3^{LZ} vector, the sequence encoding JIP3^{LZ} was amplified by PCR from a murine JIP3 template (GenomeCUBE) and cloned into the NotI/EcoRI sites of

pMW-GST3C. For CD and binding studies, we used a $JIP3^{LZ}$ construct N-terminally fused to a tryptophan residue (Trp-$JIP3^{LZ}$) for quantitation of protein concentration by UV-VIS. The pMW-GST3C-Trp-$JIP3^{LZ}$ vector was constructed as above but using a forward primer encoding an in-frame, N-terminal tryptophan residue. All inserts were sequence-verified.

Protein Production

The pMW plasmids do not contain the *lac* repressor gene or a *lac* operator sequence downstream from the T7 promoter. Proteins were produced by leaky expression in BL21(DE3) Rosetta cells. The relevant plasmid was transformed into BL21(DE3) Rosetta cells. 5 ml LB-ampicillin (100 μg/ml) cultures were inoculated from the resulting colonies and grown at 37°C for 8 hours. Each 5 ml culture was then used to inoculate a 1 L LB-ampicillin culture. The 1 L cultures were grown in 2 L baffled flasks overnight at 30°C and 185 rpm. Cultures were clarified by centrifugation and the pellets were re-suspended in cold binding buffer supplemented with cOmplete EDTA-free protease inhibitor cocktail (Roche). Re-suspended pellets were stored at -80°C until further use. Re-suspended bacteria were thawed and lysed by sonication on ice. Lysates were clarified by centrifugation.

Protein Purification

For Crystallographic Studies

The His-SUMO-$KLC2^{TPR}$ protein was purified by nickel-ion affinity chromatography. A 1 ml His-trap column (GE Healthcare) was equilibrated in binding buffer (0.5 M NaCl, 50 mM HEPES, 25 mM imidazole, 1 mM TCEP; final pH adjusted to 8.0). The clarified lysate was loaded onto the column, and unbound material washed out back to baseline with binding buffer. Bound protein was then eluted in a linear gradient of elution buffer (0.5 M NaCl, 50 mM HEPES, 500 mM imidazole, 1 mM TCEP; final pH adjusted to 8.0). Fractions were analysed by SDS-PAGE and pure fractions pooled. The his-SUMO tag was cleaved off overnight at 4°C with ULP1 protease. The $KLC2^{TPR}$ protein was separated from the his-SUMO tag by size-exclusion chromatography on a Superdex 75 16/600 column (GE Healthcare) equilibrated in 100 mM NaCl, 25 mM HEPES, 1 mM TCEP (final pH 7.5).

The GST3C-$JIP3^{LZ}$ protein was batch-purified using glutathione sepharose 4B resin (GE Healthcare). Resin was pre-equilibrated in binding buffer (0.25 M NaCl, 50 mM TRIS.HCl, 10 % glycerol, 0.1 % triton X100, 1 mM EDTA, 1 mM TCEP; final pH 7.5) and incubated in suspension with the clarified lysate for at least 4 hours at 4°C. Unbound material was removed by washing the resin 5 times in wash buffer (0.25 M NaCl, 50 mM TRIS.HCl, 1 mM TCEP; final pH 7.5). The $JIP3^{LZ}$ was obtained by cleaving the resin-bound GST3C-$JIP3^{LZ}$ protein with 3C protease overnight at 4°C, and purified by size-exclusion chromatography as described above. The pooled fractions were further purified to remove contaminating GST by anion exchange chromatography as follows. The protein was diluted 4-fold in 25 mM HEPES pH 7.0 to a final NaCl concentration of 25 mM. The diluted protein was applied to a 1 ml HiTrap Q XL column (GE Healthcare) pre-equilibrated in 25 mM HEPES pH 7.0. The pure $JIP3^{LZ}$ was collected as the flow through. The NaCl concentration in the pure protein was increased to 100 mM using a 5 M NaCl solution.

For Functional Studies

For ITC studies with the $KLC2^{TPR}$ and GST-$JIP3^{LZ}$, the high $KLC2^{TPR}$ concentrations required to use this protein as titrant necessitated a higher salt concentration than that used for crystallographic studies. The $KLC2^{TPR}$ was prepared as above for crystallographic studies but the final size exclusion chromatography step was performed in 500 mM NaCl, 25 mM HEPES, 5mM $MgCl_2$, 1 mM TCEP (final pH 7.5). The GST-$JIP3^{LZ}$ protein was bound to glutathione sepharose resin in batch as described above for the $JIP3^{LZ}$. The GST-$JIP3^{LZ}$ protein was eluted in 0.25 M NaCl, 50 mM TRIS.HCl, 1 mM TCEP, 25 mM glutathione; final pH 7.5, and purified by size exclusion chromatography on a Superdex 200 16/600 column (GE Healthcare) equilibrated in 500 mM NaCl, 25 mM HEPES, 5mM $MgCl_2$ and 1 mM TCEP (final pH 7.5).

The Trp-$JIP3^{LZ}$ protein used for CD and ITC experiments was prepared as described above for the $JIP3^{LZ}$, with the following modifications. Following 3C cleavage, contaminating GST3C was removed by incubation of the eluate with fresh, pre-equilibrated glutathione sepharose 4B resin for 2 hours at room temperature. The Trp-$JIP3^{LZ}$ was then further purified by size exclusion chromatography using a Superdex 75 16/600 column equilibrated in 100 mM NaCl, 25 mM HEPES, 1 mM TCEP (final pH 7.5). Wild-type/mutant $KLC2^{TPR}$-myc samples used for CD and ITC experiments were prepared as described above the $KLC2^{TPR}$ used in crystallographic studies. The Trp-$JIP3^{LZ}$ and $KLC2^{TPR}$-myc samples used in the fluorescence anisotropy-based binding competition assays were prepared as for those used in the CD and ITC studies, but the final size-exclusion step was performed in 100 mM NaCl, 25 mM HEPES, 5mM $MgCl_2$, 1 mM TCEP (final pH 7.5).

Crystal Structure Determination

The $KLC2^{TPR}$:$JIP3^{LZ}$ Complex

The purified $JIP3^{LZ}$ protein was concentrated in a Vivaspin column (PES membrane, 2 kDa cut-off) to a concentration of 0.63 mg/ml as quantified by a Bradford assay, and mixed with pure $KLC2^{TPR}$ (at 4.1 mg/ml) to give a final $KLC2^{TPR}$:$JIP3^{LZ}$ stoichiometry of 3:1. The complex was concentrated in a spin concentrator (Vivaspin, PES, 10 kDa cut-off) to a total protein concentration of 4.5 mg/ml, and incubated at 4°C overnight. Sitting drop crystallisation trials were set up the following day in SWISSCI MRC 3-well crystallisation plates (Jena Bioscience) using a Formulatrix NT8 crystallisation robot before storage and imaging in a Formulatrix Rock Imager at 19°C. Three-dimensional crystals grew in condition A10 of the Wizard Classic 3/4 HT96 screen (Molecular Dimensions) in a drop composed of 200 nl protein and 100 nl reservoir (20% PEG 3350, 0.2 M NaCSN); the drop ratio was further optimised to 200 nl protein plus 50 nl reservoir. Crystals were cryoprotected in 10% PEG 3350, 75 mM NaCSN, 150 mM NaCl, 37.5 mM HEPES pH 7.5 and 25% glycerol and flash frozen in liquid nitrogen. X-ray diffraction data were collected at beamline I24 of Diamond Light Source (Didcot,

Oxfordshire, UK) at a wavelength of 0.96861 Å. Data were processed using XDS (Kabsch, 2010) and programs from the CCP4 program suite (Winn et al., 2011). Phases were obtained by molecular replacement with PHASER (McCoy et al., 2007), using the crystal structures of KLC2^{TPR} (PDB: 3CEQ) and the JIP4^{LZ} (chains C and D from PDB: 2W83) (Isabet et al., 2009; Zhu et al., 2012). The structure was rebuilt in COOT (Emsley et al., 2010) and refined in BUSTER 2.10.3 (Global Phasing) to 3.2 Å resolution, with each TPR domain and the LZ domain as separate TLS groups, LSSR NCS restraints, and LSSR restraints to the higher resolution structures of the JIP4^{LZ} (PDB: 2W83) and KLC1^{TPR} (PDB: 3NF1) (Isabet et al., 2009; Zhu et al., 2012). The model was validated using the MOLPROBITY server (http://molprobity.biochem.duke.edu) (Chen et al., 2010). The final model had a MOLPROBITY score of 1.91 (100th percentile), with 96.75/0% residues in the favoured/forbidden regions of the Ramachandran plot. Figures were prepared using PYMOL (Schrodinger).

The KLC2^{TPR}:CSTN-WD2 Complex

CSTN-WD2 peptide was dissolved in KLC2^{TPR} gel filtration buffer and mixed with purified KLC2^{TPR} protein, at 2:1 (peptide:TPR) stoichiometry, giving a final KLC2^{TPR} concentration of 5.5 mg/ml. The complex was incubated at room temperature for 1 hour prior to setting up robotic crystallisation trials at 19°C. Crystals were grown in condition D1 of the JCSG Core III screen (1M sodium potassium tartrate, 0.2 M NaCl, 0.1 M imidazole pH 8.0) in a drop composed of 100 nl protein and 100 nl reservoir. This was further optimized to 0.893 M sodium potassium tartrate, 0.2 M NaCl, 0.1 M imidazole pH 8.0. Crystals were cryoprotected in reservoir plus 25% glycerol and flash frozen in liquid nitrogen. X-ray diffraction data were collected at beamline I04-1 of Diamond Light Source (Didcot, Oxfordshire, UK) at a wavelength of 0.9200 Å. Data were processed as described above. Phases were obtained by molecular replacement with PHASER (McCoy et al., 2007) using PDB: 3ZFW (Pernigo et al., 2013) as the search model. Since the search model lacked TPR1, this was modelled from the structure of the KLC1^{TPR} (Zhu et al., 2012) (PDB: 3NF1). The CSTN-WD2 peptide structure was rebuilt and the model refined in BUSTER 2.10.3 (Global Phasing) to 4.0 Å resolution with each TPR domain:peptide complex as a separate TLS group, LSSR NCS restraints, and LSSR restraints to the higher resolution structure of the KLC1 TPR domain (PDB: 3NF1) (Zhu et al., 2012). The structure was then refined in PHENIX (Adams et al., 2010) with NCS and secondary structure restraints applied. The final model had a MOLPROBITY score of 1.58, with 96.2/0% residues in the favoured/forbidden regions of the Ramachandran plot.

Isothermal Titration Calorimetry

To study the binding of the KLC2^{TPR} to GST3C-JIP3^{LZ}, the proteins were prepared by size exclusion chromatography in 0.5 M NaCl, 25 mM HEPES, 5 mM $MgCl_2$, 1 mM TCEP, final pH 7.5. ITC measurements were performed on a MicroCal iTC200 calorimeter (Malvern) at 25°C, with a differential power of 5.0 μcal/s and stirring at 750 rpm. Experiments consisted of an initial sacrificial 0.5 μl injection, followed 120 s later by 19 injections of 2 μl spread over 4 s, spaced 120 s apart. Thermograms were integrated and corrected for heats of dilution using NITPIC (http://biophysics.swmed.edu/MBR/software.html). The resulting isotherms were analysed in SEDPHAT (http://www.analyticalultracentrifugation.com/sedphat/) (Keller et al., 2012; Zhao et al., 2015). Four experiments were performed which are shown in Figures 2A–2D. The isotherms from these experiments were globally fitted with the A + B + B → AB + B → ABB model. During the fitting, the inactive fractions of A and B were fitted globally, whilst the cell and syringe concentrations and baselines of each experiment were fitted locally. The locally and globally fitted parameters are listed in Tables S1 and 2, respectively. Figures were prepared using GUSSI (http://biophysics.swmed.edu/MBR/software.html).

To study binding of the Trp-JIP3^{LZ} and FITC-CSTN-WD2 peptide to wild-type and mutant KLC2^{TPR}-myc, the recombinant proteins were prepared by size exclusion chromatography in 0.1 M NaCl, 25 mM HEPES, 1 mM TCEP, final pH 7.5. Wild-type or mutant KLC2^{TPR}-myc at 44-50 μM were placed in the cell. The Trp-JIP3^{LZ} was used as the titrant at dimer concentrations of 297-330 μM. The FITC-CSTN-WD2 peptide was used as the titrant at a concentration of 380 μM. The peptide concentration was determined by measuring the absorbance at 483 nm and using a molar extinction coefficient of 68,000 $M^{-1}cm^{-1}$. ITC experiments were performed as described above with injections spaced 120-150 s apart. Thermograms were integrated and corrected for heats of dilution using NITPIC (http://biophysics.swmed.edu/MBR/software.html). The resulting isotherms were analysed in SEDPHAT. Data for the Trp-JIP3^{LZ} as titrant were fitted with the A + B + B → AB + B → ABB model, whilst binding of the FITC-CSTN-WD2 peptide was analysed using the A + B → AB model. Between 2 and 3 experiments were performed for each titrand/titrant pair. For each titrand/titrant pair, the isotherms from the experiments were globally fitted with the relevant model. During the fitting, the inactive fractions of A and B were globally fitted, whilst the cell and syringe concentrations and baselines of each experiment were fitted locally. The results are listed in Table S2. For the experiments with the FITC-CSTN-WD2 peptide as titrant, Table S2 also lists the mean apparent binding stoichiometry value (N_{app}) for each titrand/titrant pair, defined as

$$N_{app} = \frac{1}{n}\frac{A_{KLC2}}{A_{lig}}\sum_{i=1}^{n}\frac{F_{KLC2,i}}{F_{lig,i}},$$

where $F_{KLC2,i}$ and $F_{lig,i}$ are the locally-fitted correction factors for KLC2 and ligand concentrations for experiment i, and A_{KLC2} and A_{lig} are the active fractions of KLC2 and ligand globally fitted over the n experiments. During fitting, the FITC-CSTN-WD2 peptide concentrations consistently refined to values around 30% greater than the measured value of 380 μM, with N_{app} values around 0.7 (Table S2). We ascribe this to an under-quantification of the FITC-CSTN-WD2 peptide concentration since the peptide contains a single tryptophan-acidic motif, and the concentration values for experiments with Trp-JIP3^{LZ} as ligand refined close to their measured values.

CD Spectroscopy

All CD spectra were recorded on a Chirascan CD spectrometer (Applied Photophysics) in quartz cuvettes with a 0.1 cm path length. Data were recorded at 20°C between 260 - 180 nm in 1 nm increments and a 2 nm bandwidth over an average of two scans. The $KLC2^{TPR}$-myc and Trp-$JIP3^{LZ}$ proteins were dialysed extensively into 20 mM potassium phosphate pH 7.5, 0.5 mM DTT. Spectra were collected from $KLC2^{TPR}$-myc samples at concentrations of 0.29, 0.14 and 0.086 mg/ml. Trp-$JIP3^{LZ}$ spectra were collected at 0.15, 0.074 and 0.044 mg/ml. One spectrum was recorded for each sample at each concentration and background-corrected by subtraction of a buffer-only spectrum. Corrected spectra were converted to units of mean residue ellipticity and deconvolved using the Dichroweb server (Whitmore and Wallace, 2008). $KLC2^{TPR}$-myc and Trp-$JIP3^{LZ}$ spectra were analysed using the SELCON3 and CONTIN algorithms, respectively, using reference dataset set 4 (Sreerama et al., 1999; van Stokkum et al., 1990). For each protein, the mean percentage of each secondary structure type, and the corresponding standard deviation, was calculated from the values obtained from the three concentrations. The secondary structure content for $KLC2^{TPR}$-myc and Trp-$JIP3^{LZ}$ were calculated using the $KLC2^{TPR}$:$JIP3^{LZ}$ crystal structure using the STRIDE server (http://webclu.bio.wzw.tum.de/cgi-bin/stride/stridecgi.py) (Frishman and Argos, 1995).

SEC-MALLS Experiments

Fifty microliters of $JIP3^{LZ}$ was injected onto a WTC-030S5 column, pre-equilibrated with buffer containing 25 mM HEPES pH 7.5, 0.1 M NaCl and 1 mM TCEP at a flow rate of 1 ml/min. Data were recorded using a DAWN 8+ multi-angle light scattering (LS) detector, an Optilab T-rEX differential refractive index (dRI) detector and UV absorbance (UV) detector (Wyatt Technology) and analyzed with the Astra 6.2 software package provided by the manufacturer (Wyatt Technology).

Binding Competition Experiments

The binding affinity between the FITC-labelled LFP-motif peptide and the $KLC2^{TPR}$-myc was measured essentially as described previously (Yip et al., 2016). FITC-labelled LFP motif peptide (300 nM) was incubated with 2-fold dilutions of $KLC2^{TPR}$-myc in assay buffer (0.1 M NaCl, 25 mM HEPES, 5 mM $MgCl_2$, 1 mM TCEP, final pH 7.5) in triplicate in 384-well plates, for 1 hour at room temperature. Fluorescence measurements were recorded on a TecanSpark plate reader at excitation and emission wavelengths of 488 nm and 535 nm respectively. Fluorescence anisotropy values were calculated at each $KLC2^{TPR}$-myc concentration and the data were fitted with a fixed-slope dose response curve in Prism (GraphPad). This gave an affinity of 37 μM, similar to that found previously (Yip et al., 2016). For competition experiments, 2-fold serial dilutions of unlabelled CSTN-WD1 peptide or Trp-$JIP3^{LZ}$ were incubated with the FITC-labelled LFP motif peptide (300 nM) and a fixed concentration of $KLC2^{TPR}$-myc (21 μM). A K_I value was obtained for the CSTN-WD1 peptide data by fitting the anisotropy values A with following equation using Prism (Graphpad):

$$A = A_{min} + (A_{max} - A_{min}) \times \frac{\left([KLC] - [CSTN] - K_I + \sqrt{(K_I + [KLC] + [CSTN])^2 - 4[KLC][CSTN]}\right)}{2K_{LFP} + [KLC] - [CSTN] - K_I \sqrt{(K_I + [KLC] + [CSTN])^2 - 4[KLC][CSTN]}}$$

where A_{min} and A_{max} are the minimum and maximum anisotropy values, [KLC] and [CSTN] are the total concentrations of $KLC2^{TPR}$-myc and calsyntenin-1 peptide, respectively, and K_{LFP} is the dissociation constant for the FITC-LFP motif peptide binding to the $KLC2^{TPR}$-myc (37 μM; see above). Two independent experiments were performed with the Trp-$JIP3^{LZ}$. One experiment was performed for the CSTN-WD1 peptide. Graphs in Figure 4B show data from a single representative experiment.

Bioinformatic Sequence Analyses

Two datasets were constructed from available genomic and transcriptomic data. Combined, these two datasets ensured dense sampling of bilaterian and non-bilaterian species. Dataset 1 was constructed using the OMA Orthology database (Altenhoff et al., 2015) and contains protein-coding sequences at the amino acid level from 59 Bilaterian genomes (42 *Chordata*, 10 *Arthropoda*, 3 *Nematoda*, 2 *Annelida*, 1 *Playthelminthes* and 1 *Mollucsa*) and 3 non-Bilaterian genomes (1 *Porifera*, 1 *Ctenophora* and 1 *Cindaria*). Dataset 2 was assembled from Simion et al. (Simion et al., 2017) and consisted of 3 *Ichthyosporea*, 14 *Porifera*, 15 *Ctenophora* and 10 *Cnidaria*, including 15 recently sequenced non-bilateria. Searches were carried out using BLASTp (Altschul et al., 1997) for Dataset 1 and TBLASTN (Gertz et al., 2006) for Dataset 2 with human JIP3 (NP_055948.2) and KLC2 (NP_001305663.1) as the query sequences. The KLC and JIP3 residues at the interfaces depicted in Figure 5B were identified as described above. The motifs were defined as follows. JIP3 binding site on KLC: residues 200-201, 204, 208, 213, 215-216, 219-20, 223 and 227. Tryptophan-acidic cargo binding site on KLC: residues 244-245, 248, 251, 263, 270, 283- 284, 286-287, 290-291, 294, 305, 312, 325, 329, 332-333, 335-336. TPR1 dimerisation site: 197, 200-201, 204, 207-208, 213, 215-216, 219-220, 223. KLC binding site on JIP3: residues 432-434, 436-445 and 448-449. The conservation of these residues, and the KLC2 LFP motif (residues 167-169), was assessed from the resulting pairwise alignments. In general, strict conservation of all residues was required for a motif to be classified as conserved in a given homolog. The exception to this was the KLC-binding region of JIP3. Here, sequence variation at residues 432-433, 439, 442 and 446-447 was allowed, which was based on comparison of human, *D. melanogaster* and *C. elegans* JIP3/4 homologs and prior knowledge of binding in these species (Bowman et al., 2000; Byrd et al., 2001; Nguyen et al., 2005). The full list of results is tabulated in Tables S3 and S4. The results were then compared with the current canonical species phylogeny for Metazoa (Simion et al., 2017).

Structural Analyses

Analysis of Protein:Protein Interfaces

Buried surface areas were calculated using program AREAIMOL from the CCP4 suite (Winn et al., 2011). Residues at protein:protein interfaces were identified using program CONTACT from the CCP4 suite with a 4.2 Å interatomic distance cut off. Any amino acid with at least one atom within the cut-off distance of an atom from the target protein was defined as being at the interface. Putative hydrogen bonds were assigned using a cut off distance of 3.4 Å between donor and acceptor N/O atoms across the interface.

KLC2^{TPR} Binding to ARF6-bound JIP4^{LZ}

The KLC2^{TPR}:JIP3^{LZ} complex was superposed onto the crystal structure of the ARF6:JIP4^{LZ} complex (Isabet et al., 2009) (PDB: 2W83), via residues 433-450 from JIP3^{LZ} chains C and D and the equivalent residues in the JIP4^{LZ} using LSQKAB from the CCP4 suite (Winn et al., 2011) (r.m.s.d. of 0.4 Å between equivalent C_α atoms). Full length ARF6 contains an N-terminal myristoylation group on residue 2 and an amphipathic helix (residues 2-11), both of which were absent from the ARF6:JIP4^{LZ} crystal structure. The thickness of the membrane and positioning of the ARF6 amphipathic helix along the bilayer normal in Figure 1E are taken from on neutron scattering experiments of DOPC bilayers in complex with an amphipathic helix (Hristova et al., 1999).

Crystal Packing Analysis

For each copy of the KLC^{TPR} in the crystallographic asymmetric unit, the neighbouring TPR domains inside the crystal were identified in COOT (Emsley et al., 2010) using a cut-off of 10 Å between C_α atoms. The packing models for each independent TPR domain from the various crystal structures were superposed via the subunit of interest onto PDB: 3NF1, using SUPERPOSE (Krissinel and Henrick, 2004) from the CCP4 program suite (Winn et al., 2011). This gave pairwise r.m.s.d. values of 1.7-2.6 Å for 226-250 equivalent C_α atoms. The TPR1:TPR1 interfaces were compared by pairwise superposition of the dimers via TPR1 using SUPERPOSE from the CCP4 suite (Krissinel and Henrick, 2004; Winn et al., 2011). TPR1 was defined as residues 195-236 for KLC2 and residues 210-251 for the KLC1 TPR domain. The sequence register in the PDB: 5FJY TPR1s was shifted by one residue relative to that in the other structures. The TPR1s in PDB: 5FJY were modelled from a 4 Å resolution electron density map that showed very few features for amino acid side-chains (Yip et al., 2016). Comparison of PDB: 5FJY with the highest resolution structure available (the KLC1^{TPR}, solved to 2.8 Å resolution (Zhu et al., 2012); PDB: 3NF1) showed that the TPR1 polypeptide chain conformations were very similar (r.m.s.d. values of 0.7-0.8 Å between 82 structurally equivalent TPR1 C_α atoms). Taken together with the fact that the KLC1 and KLC2 TPR1s are 100% identical in amino acid sequence, we interpreted this discrepancy as being due to difficulties in modelling the PDB: 5FJY TPR1s into the original electron density map, rather than a difference in structure of the TPR1:TPR1 interfaces between these two crystals.

QUANTIFICATION AND STATISTICAL ANALYSIS

Information on the number of experiments performed and definitions of errors and means can be found in the relevant sections of the methods and the table/figure legends.

DATA AND SOFTWARE AVAILABILITY

The accession numbers for the coordinates and structure factors for the KLC2^{TPR}:JIP3^{LZ} and KLC2^{TPR}:CSTN-WD2 crystal structures reported in this paper are PDB: 6EJN and PDB: 6F9I respectively.

Structure, Volume *26*

Supplemental Information

Insights into Kinesin-1 Activation

from the Crystal Structure of KLC2 Bound to JIP3

Joseph J.B. Cockburn, Sophie J. Hesketh, Peter Mulhair, Maren Thomsen, Mary J. O'Connell, and Michael Way

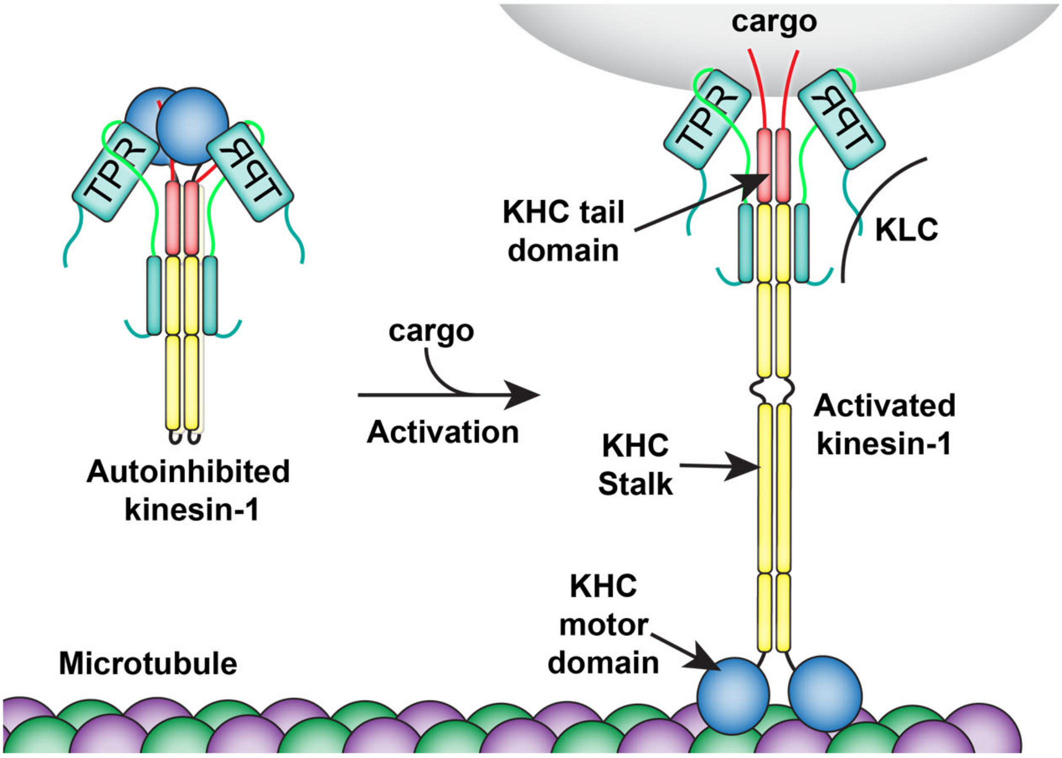

Figure S1

Figure S1. Related to Figure 1. Overview of kinesin and JIP3

Schematic of the kinesin heterotetramer in the autoinhibited and activated states.

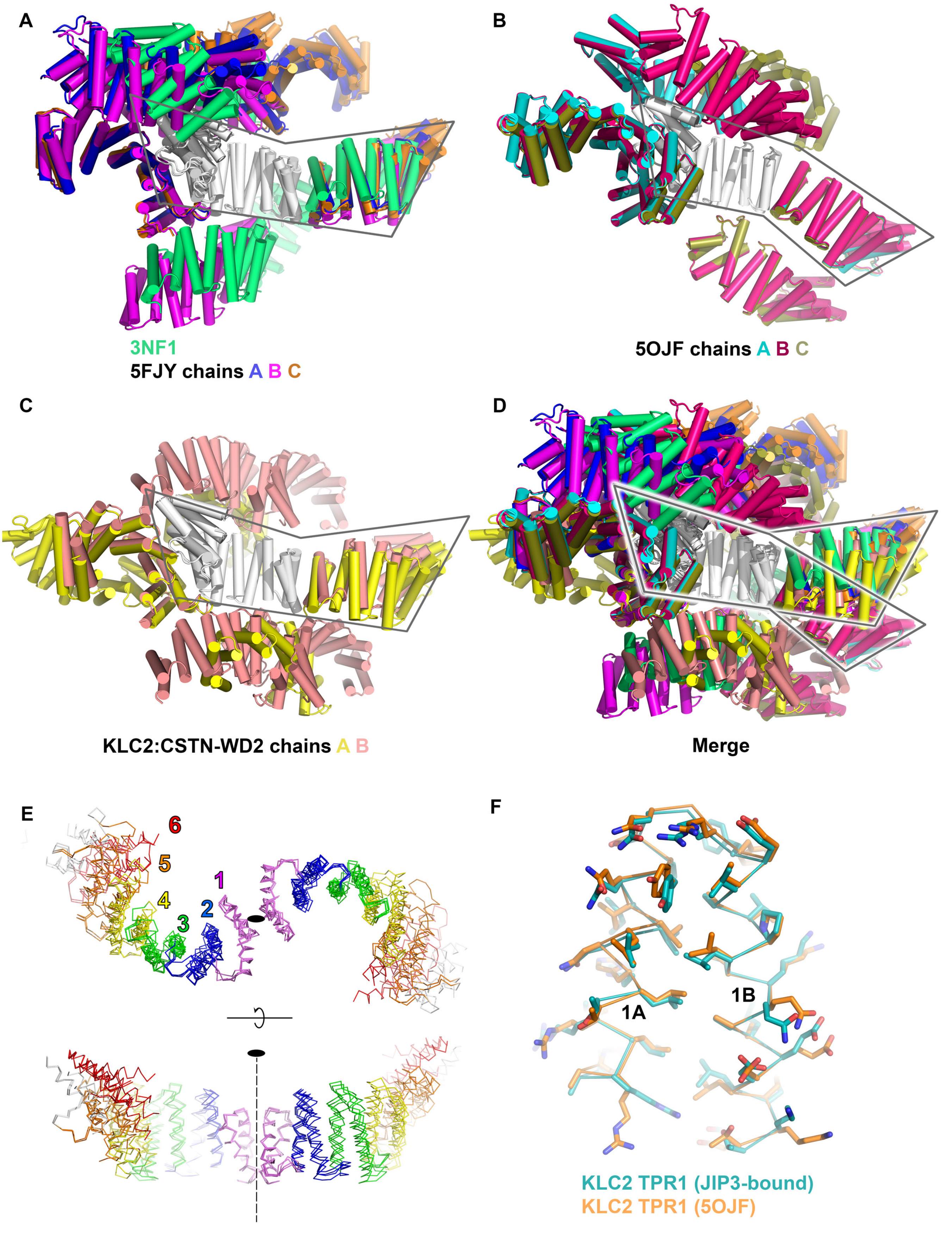

A
3NF1
5FJY chains A B C
B
5OJF chains A B C
C
KLC2:CSTN-WD2 chains A B
D
Merge
E
6
5
4
3
2
1
F
1A
1B
KLC2 TPR1 (JIP3-bound)
KLC2 TPR1 (5OJF)

Figure S2

Figure S2. Related to Figure 6. Dimerisation of KLC TPR domains via TPR1.

(**A-D**) Crystal packing interactions in (**A**) 3NF1 and 5FJY, (**B**) 5OJF, (**C**) our KLC2:CSTN-WD2 structure, and (**D**) all four structures. The environments of the crystallographically independent subunits (white) are superposed, with neighbours of 5FJY chains A/B/C coloured blue/magenta/orange; neighbours of 3NF1 chain A - green; neighbours of 5OJF chains A/B/C coloured cyan/hot pink/olive; and neighbours of KLC2:CSTN-WD2 chains A/B - pink/yellow. The conserved dimers are outlined.

(**E**) All 4 dimers from 3NF1 5FJY and our KLC2:CSTN-WD2 structure, coloured by TPR, superposed via TPR1 (dimer 2-fold axis – dashed line with oval).

(F) Superposition of KLC2 TPR1 from our KLC2:JIP3 complex (teal) and KLC2 TPR1 from 5OJF (orange), demonstrating very similar main-chain and side-chain conformations in these two structures.

Table S1. Related to Table 2 and Fig. 2. Locally-fitted parameters for ITC experiments with $KLC2^{TPR}$ and $GST\text{-}JIP3^{LZ}$

**Expt.** [a]	**Cell**	**Syringe**	**Nominal cell conc. (μM)**	**Nominal syringe conc. (μM)**	**Fitted cell conc. (μM)**	**Fitted syringe conc. (μM)**	**Fitted baseline (kCal/mol)**
1	$KLC2^{TPR}$	$GST\text{-}JIP3^{LZ}$	38.0	332	36.5	392.5	0.45
2	$KLC2^{TPR}$	$GST\text{-}JIP3^{LZ}$	71	480	70.8	589.6	0
3	$GST\text{-}JIP3^{LZ}$	$KLC2^{TPR}$	47.0	418	49.5	481.5	0
4	$GST\text{-}JIP3^{LZ}$	$KLC2^{TPR}$	22.0	392	19.8	397.2	-0.32

[a] Results from experiments 1-4 are shown in Fig. 2A-D respectively

Table S2. Related to Fig. 3. Thermodynamics of the Trp-JIP3(LZ) domain and the FITC-CSTN-WD2 peptide binding to wild-type and mutant KLC2(TPR) domains

**KLC2 mutant**	**Ligand**	***K*** **(μM^{-1})**[a]	**Δ*H*** **(kCal/ mol)**	**-*T*Δ*S*** **(kCal/ mol)**	**Fitted concentration correction factors**[b]		**Fitted active fractions**[c]		N_{app}[d]	***n***[e]
					**KLC2**	**Ligand**	**KLC2**	**Ligand**		
WT	LZ	0.119[f]<br>0.129[g]	-19.667[f]<br>-4.719[g]	12.744[f]<br>-2.254[g]	0.98 ± 0.01	1.04 ± 0.06	1.0	1.0	N/A	3
R312E	LZ	0.229[f]<br>0.166[g]	-21.331[f]<br>-3.027[g]	14.019[f]<br>-4.093[g]	0.89 ± 0.08	0.99 ± 0.03	1.0	1.0	N/A	3
WT	CSTN	0.820	-4.305	-3.762	0.92 ± 0.18	1.32 ± 0.09	0.97	1.0	0.67 ± 0.08	2
Y208A	LZ	N/D[e]	N/D	N/D	N/D	N/D	N/D	N/D		2
Y208A	CSTN	0.541	-3.358	-4.463	0.98 ± 0.07	1.31 ± 0.04	0.90	1.0	0.67 ± 0.07	2
T200D	LZ	N/D	N/D	N/D	N/D	N/D	N/D	N/D		2
T200D	CSTN	0.845	-4.174	-3.911	0.96 ± 0.03	1.34 ± 0.03	1.0	1.0	0.71 ± 0.03	3
L220Y	LZ	N/D	N/D	N/D	N/D	N/D	N/D	N/D		2
L220Y	CSTN	0.739	-3.902	-4.104	0.92 ± 0.00	1.30 ± 0.02	1.0	1.0	0.71 ± 0.01	2

[a] Macroscopic association constant

[b] Locally-fitted correction factors for KLC2 and ligand concentrations during fitting of the relevant model (mean ± standard deviation of the locally fitted values in the *n* experiments)

[c]Active fractions of KLC2 or ligand (globally-fitted over the *n* experiments in the dataset during fitting of the relevant model)

[d]Apparent binding stoichiometry (see Methods; mean ± standard deviation of values from the *n* experiments)

[e]Number of experiments

[f,g]Parameters for $KLC2^{TPR}$ binding to the first and second sites on the $Trp\text{-}JIP3^{LZ}$ dimer, respectively.

[h]Not determined

Table S3. Related to Figure 5. Conservation of the JIP3 KLC-binding site, and KLC cargo binding sites, LFP motif, and TPR1 dimerisation site in Bilaterian species*

Species	KLC binding site on JIP3	JIP3 binding site on KLC	Tryptophan-acidic cargo binding site	KLC LFP motif	KLC TPR1 dimerisation interface
**Chordata**					
A. platyrhynchos	✓	✓	✓	✓	✓
A. carolinensis	✓	✓	✓	✓	✓
A. mexicanus	✓	✓	✓	✓	✓
B. floridae	✓	✓	✓	✓	✓
G. gallus	✓	✓	✓	✓	✓
C. hoffmanni	✓	✗	✗	✓	✗
C. intestinalis	✓	✓	✓	✗	✓
C. savignyi	✓	✓	✓	✗	✓
D. rerio	✓	✓	✓	✓	✓
D. novemcinctus	✓	✓	✓	✗	✓
E. telfairi	✓	✓	✓	✓	✓
F. albicollis	✓	✓	✓	✓	✓
G. morhua	✗	✓	✓	✓	✓
G. aculeatus	✓	✓	✓	✓	✓
H. sapiens	✓	✓	✓	✓	✓
L. chalumnae	✓	✓	✓	✓	✓
L. oculatus	✓	✓	✓	✓	✓
L. africana	✓	✓	✓	✓	✓
M. eugenii	✓	✓	✓	✓	✓
M. gallopavo	✓	✓	✓	✓	✓
M. domestica	✓	✓	✓	✓	✓
M. musculus	✓	✓	✓	✓	✓
M. lucifugus	✓	✓	✓	✓	✓
N. leucogenys	✓	✓	✓	✓	✓
O. niloticus	✓	✓	✓	✓	✓
O. anatinus	✓	✓	✓	✓	✓
O. latipes	✓	✓	✓	✓	✓
O. garnettii	✓	✓	✓	✓	✓
P. sinensis	✓	✓	✓	✓	✓
P. marinus	N/A	✓	✓	✓	✓
S. scrofa	✓	✓	✓	✓	✓
P. formosa	✓	✓	✓	✓	✓
P. capensis	✓	✓	✓	✓	✓
R. prolixus	N/A	✓	✓	✓	✓
S. harrisii	✓	✓	✓	✓	✓
S. araneus	✓	✓	✓	✗	✓
S. purpuratus	✓	✓	✓	✗	✓
T. guttata	✓	✓	✓	✓	✓

T. rubripes	✗	✓	✓	✓	✓
T. nigroviridis	✓	✓	✓	✓	✓
X. tropicalis	✓	✓	✓	✓	✓
X. maculatus	✓	✓	✓	✓	✓
**Arthropoda**					
A. aegypti	✗	✓	✓	✓	✓
A. gambiae	✓	✓	✓	✓	✓
D. plexippus	✓	✓	✓	✓	✓
D. pulex	✓	✓	✓	✓	✓
D. melanogaster	✓	✓	✓	✓	✓
N. vitripennis	N/A	✓	✓	✓	✓
S. maritima	✓	✓	✓	✗	✓
T. urticae	✓	✓	✓	✗	✓
T. castaneum	✓	✓	✓	✓	✓
Z. nevadensis	✓	✓	✗	✓	✓
**Nematoda**					
C. briggsae	✓	✓	✓	✗	✓
C. elegans	✓	✓	✓	✗	✓
O. volvulus	✓	✓	✓	✗	✓
**Annelida**					
C. teleta	✓	✓	✓	✗	✓
H. robusta	✓	✓	✓	✗	✓
**Mollusca**					
L. gigantea	✓	✓	✓	✗	✓
**Platyhelminthes**					✓
S. mansoni	✓	✓	✓	✗	✓

*Key to symbols:
✓ - conserved (see Materials and Methods)
✗ - not conserved
N/A - no homology detected

Table S4. Related to Figure 5. Conservation of the JIP3 KLC-binding site, and KLC cargo binding sites, LFP motif, and TPR1 dimerisation site in non-Bilaterian species*

Species	KLC binding site on JIP3	JIP3 binding site on KLC	Tryptophan-acidic cargo binding site	KLC LFP motif	KLC TPR1 dimerisation interface
**Ichthyosporea**					
A. whisleri	N/A	N/A	✗	N/A	N/A
C. fragrantissima	N/A	N/A	N/A	N/A	N/A
P._gemmata	N/A	N/A	N/A	N/A	N/A
**Porifera**					
A. queenslandica	N/A	✗	✗	✗	✗
A. vastus	✗	✗	✗	✗	✗
C. nucula	✗	N/A	✗	✗	✗
C. candelabrum	✗	✓	✓	✗	✗
E. muelleri	✗	✗	✗	✗	✗
G. compressa	✗	✓	✗	✗	✓
I. fasciculata	N/A	✗	✗	✗	✗
L. nivea	✗	✓	✗	✗	✓
O. carmela	✗	✗	✗	N/A	✗
P. ficiformis	✗	✗	✗	✗	✗
P. jani	✗	✓	✓	N/A	✗
P. spinifera	✗	✗	✗	✗	✗
S. lacustris	N/A	✗	✗	N/A	✗
S. coactum	✗	✓	✗	✗	✓
Ctenophora					
B. abyssicola	N/A	✓	✗	✗	✓
B. infundibulum	N/A	✗	✗	✗	✗
C. veneris	N/A	✗	✗	N/A	N/A
C. astericola	N/A	✗	✓	N/A	✗
C. meteoris	N/A	✓	✓	✗	✓
Ctenophora_sp3_A	N/A	N/A	N/A	N/A	N/A
D. glandiformis	✗	✗	✗	✗	✗
E. dunlapae	N/A	✓	✗	✗	✓
L. pancerina	N/A	✓	✓	✗	✓
M. leidyi_A	N/A	✓	✗	✗	✓
M. leidyi	✗	✓	✓	✗	✓
P. pileus_A	N/A	✓	✓	N/A	✓
P. pileus	N/A	✓	✓	✗	✓
V. multiformis_A	N/A	N/A	N/A	N/A	N/A
V. multiformis	✗	✓	✓	✗	✓
**Cnidaria**					
A. caribbeana	✗	✓	✗	✗	✓
C. sowerbyi	N/A	✓	✓	✗	✓
H. polyclina	✗	✓	✗	✗	✓
L. tetraphylla	✗	✓	✓	✗	✓

L. campanulata	N/A	✓	✗	✗	✓
M. auretenra	N/A	✓	✗	N/A	✓
N. vectensis	✗	✓	✓	✗	✓
P. noctiluca	N/A	✓	✗	N/A	✓
P. rubra	N/A	N/A	N/A	N/A	N/A
P. pennacea	✗	✓	✗	N/A	✗

*Key to symbols:
✓ - conserved (see Materials and Methods)
✗ - not conserved
N/A - no homology detected

Table S5. Related to Figure 6. Summary of crystal structures containing full-length KLC TPR domain

Construct	Crystallisation conditions	Space group	Unit cell parameters a, b, c (Å) α, β, γ (°)	# copies[1]	PDB entry
**murine KLC2 (191-480) + CSTN-WD2 peptide**	0.893 M sodium potassium tartrate<br>0.2M sodium chloride<br>0.1 M imidazole pH 8.0	P 3_1 2 1	75.44, 75.44, 303.36<br>90, 90, 120	2	6EJO
**murine KLC2 (161-480)**	0.1 M sodium cacodylate, pH 6.5<br>0.3 M sodium malonate<br>8% (wt/vol) PGA-LM.	C2	148.70, 86.28, 111.74<br>90, 98.4, 90	3	5FJY
**human KLC1 (205-495)[2]**	2.0 M ammonium sulfate<br>20% ethylene glycol<br>0.1 M Bis-Tris Propane pH 7.0	P 3_1 2 1	74.7, 74.7, 156.2<br>90, 90, 120	1	3NF1
**murine KLC2 (194-479)**	0.5 M sodium postassium phosphate pH 7.5<br>12% peg 3350<br>1 mM sarcosine	C2	97.62, 116.47, 108.11<br>90, 99.51, 90	3	5OJF

[1] per crystallographic asymmetric unit
[2] equivalent to murine KLC2 residues 190-480
